# Protonation Sites, Tandem Mass Spectrometry and Computational Calculations of *o*-Carbonyl Carbazolequinone Derivatives

**DOI:** 10.3390/ijms17071071

**Published:** 2016-07-05

**Authors:** Maximiliano Martínez-Cifuentes, Graciela Clavijo-Allancan, Pamela Zuñiga-Hormazabal, Braulio Aranda, Andrés Barriga, Boris Weiss-López, Ramiro Araya-Maturana

**Affiliations:** 1 Programa Institucional de Fomento a la Investigación, Desarrollo e Innovación, Universidad Tecnológica Metropolitana, Las Palmeras 3360, Casilla 9845, Santiago 7800003, Chile; 2 Departamento de Química, Facultad de Ciencias, Universidad de Chile, Las Palmeras 3425, Casilla 653, Santiago 7800003, Chile; gracielaclavijo@gmail.com (G.C.-A.); pamela.zuniga@ug.uchile.cl (P.Z.-H.); braulio.aranda.z@gmail.com (B.A.); bweiss@uchile.cl (B.W.-L.); 3 Unidad de Espectrometría de Masas, Facultad de Ciencias Químicas y Farmacéuticas, Universidad de Chile, Santos Dumont 964, Casilla 233, Santiago 8380494, Chile; anbarr@gmail.com; 4 Instituto de Química de Recursos Naturales, Universidad de Talca, Av. Lircay s/n, Casilla 747, Talca 3460000, Chile

**Keywords:** quinones, mass spectrometry, carbazole, DFT, QCISD

## Abstract

A series of a new type of tetracyclic carbazolequinones incorporating a carbonyl group at the ortho position relative to the quinone moiety was synthesized and analyzed by tandem electrospray ionization mass spectrometry (ESI/MS-MS), using Collision-Induced Dissociation (CID) to dissociate the protonated species. Theoretical parameters such as molecular electrostatic potential (MEP), local Fukui functions and local Parr function for electrophilic attack as well as proton affinity (PA) and gas phase basicity (GB), were used to explain the preferred protonation sites. Transition states of some main fragmentation routes were obtained and the energies calculated at density functional theory (DFT) B3LYP level were compared with the obtained by ab initio quadratic configuration interaction with single and double excitation (QCISD). The results are in accordance with the observed distribution of ions. The nature of the substituents in the aromatic ring has a notable impact on the fragmentation routes of the molecules.

## 1. Introduction

Quinones are a class of compounds with high structural diversity and widely present in nature [1,2,3]. Some of them perform essential roles in the respiratory chain of cells [4]. In addition, quinones are considered a privileged scaffold in medicinal chemistry as anticancer [5,6], antifungal [7,8], and antiparasitic drugs [9]. Moreover, it has been found that carcinogenic polyaromatic quinones are generated in air suspended particulate by oxidation of polycyclic aromatic hydrocarbons [10]. Interestingly, some quinones have also been found in interstellar dust particles [11]. Quinones also have relevance in industrial applications such as dyes [12], in biodegradation of priority pollutants [13] and more recently in energy storage applications [14].

In addition, the carbazole motif is present in several biologically active molecules [15,16]. For example, 1,4-Carbazolequinones have attracted interest as anticancer compounds [17,18]. The alkaloid murrayaquinone A, and a number of analogs that contain this moiety, have shown promising cytotoxicities [19]. Also calothrixin B shows a high in-vitro cytotoxicity against HeLa cancer cells, by interaction with human topoisomerase I [20] and by generation of reactive oxygen species [21].

On the other hand, mass spectrometry (MS) has been demonstrated to be a very valuable tool in chemistry and life science research [22]. The small amount of substance necessary for analysis, when compared with other techniques, confers MS a privileged site in structural analysis [23]. The fragmentation patterns of different types of molecules are one of the most useful data for structure elucidation of unknown compounds [24,25]. The analysis of quinones has been performed using diverse methodologies such as conventional gas chromatography-electron impact-mass spectrometry (GC/EI-MS) [26], matrix-assisted laser desorption/ionization time-of-flight (TOF) mass spectrometry [27], electrospray ionization mass spectrometry (ESI-MS) [28,29,30], atmospheric pressure chemical ionization mass spectrometry [31], two-step laser desorption/post-photoionization mass spectrometry (L2MS/PIMS) [32], infrared laser desorption/tunable synchrotron vacuum ultraviolet (VUV), photoionization TOF mass spectrometry [33], and, more recently, desorption electrospray ionization (DESI), developed almost twenty years after matrix-assisted laser desorption/ionization technique (MALDI) [34].

The fragmentation mechanisms of some quinones, especially substituted 1,4-naphthoquinones [35,36,37] and substituted anthraquinones [37,38] have been previously described. 

MS/MS experiments commonly lead to different routes of fragmentation of the molecule, depending on the thermodynamic and kinetic phenomena associated with the intermediates species formed during the fragmentation process. The study of these gas-phase intermediaries is not an easy task and the assistance of computational chemistry is a very useful and powerful tool to achieve this goal [39,40]. Some MS studies of quinones have been assisted in this way. For example, the role of the chain of 2-(acylamino)-1,4-naphthoquinones in the fragmentation of the protonated species generated by electrospray ionization, and in the fragmentation analysis of lapachol [30,41]. Also, the atoms in molecules theory has been used in the mass spectra analysis of 1,4-naphthoquinone derivatives, and to explain the generation of anion radicals of these class of compounds by ESI-MS [42,43].

For many years, we have been interested in quinones and hydroquinones as antitumor and antifungal agents [8,44,45,46,47,48], and in their unequivocal structural characterization using nuclear magnetic resonance (NMR) [49,50,51] and MS [52] techniques. Particularly interesting is the presence of a *o*-carbonyl group attached to the quinone/hydroquinone ring [47,53,54], which can affect both, the electronic structure of the molecule as well as the possibility to form an intramolecular hydrogen bond (intraHB) [55].

**Figure 1:**
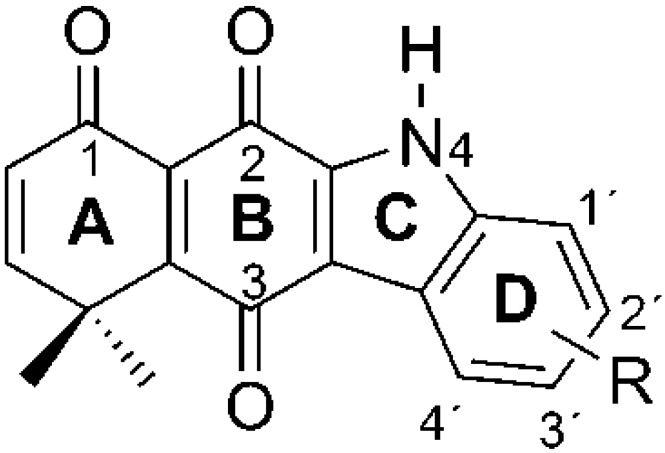
Structure of *o*-carbonyl carbazolequinones.

In this work, we report four new compounds, as an example of a new structural type of *o*-carbonyl quinone derivatives, with different substituents on ring D (Figure 1). Their gas-phase dissociation by electrospray ionization technique is also presented. In order to assist with the interpretation of the experimental results, density functional theory (DFT) and ab-initio calculations, were performed. 

## 2. Results and Discussion

### 2.1. Synthesis of Carbazolequinones

**Figure 2:**
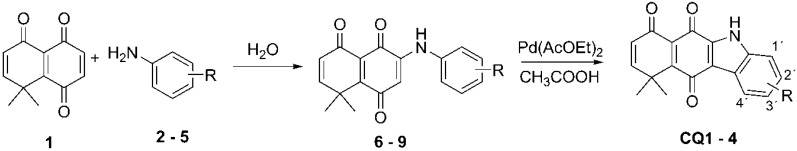
Synthetic route to obtain *o*-carbonylcarbazolquinones **CQ1** to **CQ4**.

*o*-carbonylanilinoquinones **6**–**9** were synthesized following the previously reported on water green procedure [53]. These compounds were used as starting products in an oxidative coupling with palladium acetate, under nitrogen atmosphere, generating the corresponding new *o*-carbonyl carbazolequinones **CQ1**–**CQ4** (Figure 2), following a procedure similar to one described before [56].

We observed that **CQ1,** unsubstituted in the aromatic ring, is obtained in 52% yield for the cyclization reaction. This yield increases to 77% with the 2-methyl substituted compound (**CQ2**) and decreased to 23% and 39% for compounds **CQ3** and **CQ4** (4–Br and 4–COOEt substituted, respectively), suggesting the importance of the substituent in the aromatic ring for the reactivity in this kind of cyclization reaction. 

### 2.2. Determination of Most Favorable Protonation Site

**Figure 3:**
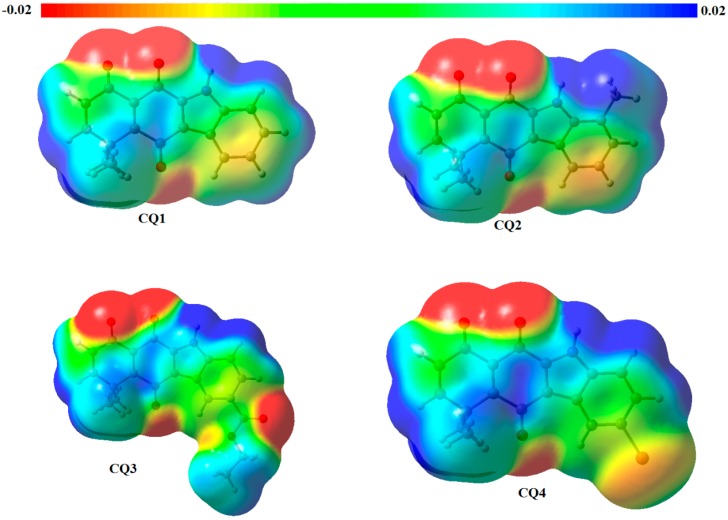
Molecular electrostatic potential maps (MEP) for *o*-carbonylcarbazolequinones **CQ1** to **CQ4**. Red color indicates high electron density and blue color low electron density.

These molecules present four to six possible protonation sites corresponding to oxygen and nitrogen atoms. Our initial approach to study the most favorable protonation site was to calculate the static properties in the neutral ground state of the molecules. The calculations of optimized structures were carried out at DFT B3LYP/6-31G(d,p) level (Cartesian coordinates for all optimized structures can be found in Tables S1–S82 (supplementary materials)). Then, molecular electrostatic potential (MEP) plots (Figure 3) were obtained, which allows a qualitative determination of the most favorable protonation site.

The MEPs plot for all CQs show a strong basic region (red color) on oxygen atoms 1 and 2, and a weak basic region on oxygen 3. Additionally, **CQ3** shows a basic region on the carbonyl ester oxygen 5′. On the other hand, nitrogen 4 does not exhibit basic character, possibly due to an efficient delocalization of the non-bonded electrons through the rest of the aromatic ring. Judging by the color (blue), it is clearly an electron deficient region. 

Another way to assess the most favorable site of protonation involves obtaining local Fukui functions for electrophilic attack (***f*^−^**) [57] and the recently developed local Parr function for electrophilic attack (***P*^−^**) [58]. The values of these parameters are displayed in Table 1. Both parameters are a quantitative measurement of the local nucleophilic reactivity and therefore, the most favorable protonation site. The new parameter ***P*^−^** has been invocated to be more suitable for polar reactions than local Fukui function. In this study, both parameters were compared.

In all **CQ**s, both parameters, ***f*^−^** and ***P*^−^**, show that the most reactive site for an electrophilic attack, towards which a proton can diffuse and eventually attach, is oxygen 1. However, according to ***P*^−^**, nitrogen 4 is shown to be the second most favorable site to attach the proton, unlike ***f***^−^, which indicates that O2 is the second most favorable site, in agreement with MEP plots. The electrostatic potential reflects the hard reactivity [59]. On the other hand, local descriptors from conceptual DFT, such as ***f*^−^** and ***P*^−^**, are representative of the soft reactivity, which have a greater orbital influence [59]. Since the intermolecular protonation reaction is mainly an electrostatically controlled phenomenon, it is reasonable to consider the results that agree with those given by MEPs plots. Additionally, these results show that DFT local descriptors should be carefully used for this kind of studies. 

Thermodynamic parameters are key tools to study and accurately determine the most favorable protonation site. For instance, the protonation sites of 1,4-benzoquinone (1,4-Bq) have been studied through experimental work and theoretically determined by proton affinity (PA) [60]. The results showed that oxygen atoms were the most favorable sites for protonation, by around 50 kcal·mol^−1^ relative to the quinone ring carbons. Experimental PA of 1,4-benzoquinone was 191.4 kcal·mol^−1^ [60]. In our case, we calculated thermodynamic parameters such as proton affinity (PA) and gas-phase basicity (GB) [60,61,62], for the protonation of all oxygen and nitrogen atoms in the molecules. The results for all protonation sites are included in the supplementary materials (Tables S83–S88). Both PA and GB (Table 2) show that the protonation on O1 and O2 are energetically more favorable than other sites, giving both the same value in all cases. Comparison of PA from these CQs with PA from 1,4-Bq shows an increase of around 50 kcal·mol^−1^ for CQs, indicating that the effect of the fused carbazole moiety on BQ favors the protonation.

However, given that the above calculations were carried out without considering the activation of the ion, it is possible that additional proton migration, induced by collisions, can occur following the initial protonation step [63].

### 2.3. Fragmentation Pathways of Carbazolquinone Derivatives 

The main fragment ions observed in the ESI-MS analysis of carbazolequinones **CQ1**–**4** are listed in Table 3. A first overview shows some differences in the behavior of **CQ1** and **CQ2**, both sodic and potassic adducts, were observed, beside the protonated species. For **CQ3** the potassic specie was not observed, while for **CQ4** only the protonated specie was observed. For **CQ4** the presence of bromide was observed by a peak with *m*/*z* 370.

Protonated species were selected and dissociated to obtain fragmentation patterns for all compounds. 

**Scheme 1:**
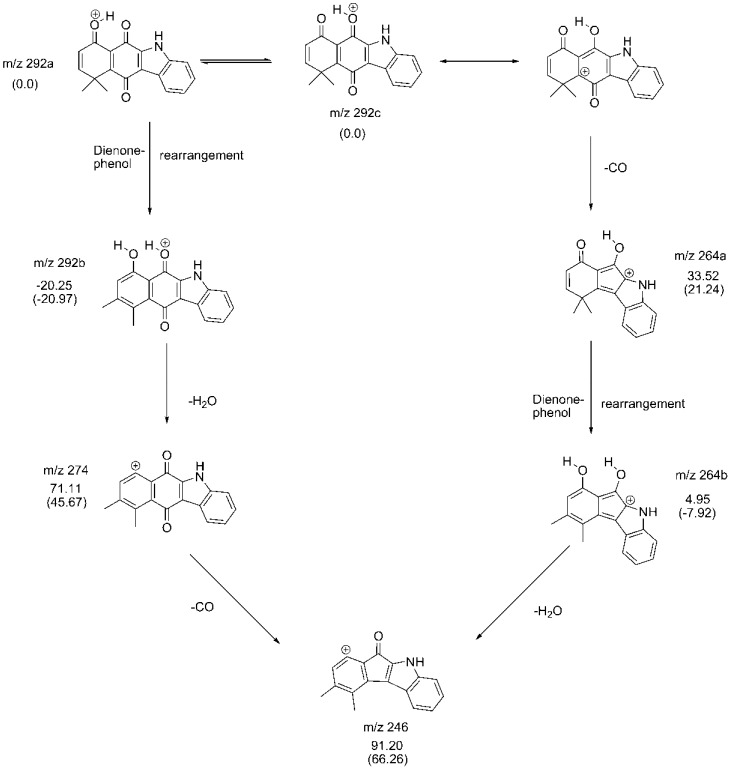
Fragmentation patterns for water and CO losses from protonated **CQ1**.

Table 4 lists the main fragments observed in the ESI-MS^n^ analysis of carbazolequinones. Starting from the initial molecular ions, we found, as a common fragmentation pattern, the water and carbon monoxide losses. In Scheme 1, a plausible mechanism representative of this fragmentation is presented for **CQ1**. On the other hand, the spectrum shows two unusual fragments with *m*/*z* 144 (100%) and *m*/*z* 149 (40%) that will be analyzed later.

Theoretical calculations were performed in selected structures and mechanisms. Enthalpies and Gibbs free energies are relative to initial molecular ion in all schemes (Gibbs free energies are presented in parentheses in all cases). From the initial protonated molecular ion at *m*/*z* 292, we propose a dienone-phenol rearrangement to achieve the water loss, giving a *m*/*z* 274 ion. CO loss should occur via ring contraction, to give the *m*/*z* 264 ion. We consider the loss of CO in the carbonyl oxygen where the protonation is less probable, according to the results in Section 3.2. Whereas water loss requires two steps, CO loss occurs directly through one step. Enthalpy and free energy from DFT calculations show that CO loss is energetically more favored. This proposition is in agreement with the percentage observed for *m*/*z* 264 (100%) and *m*/*z* 274 (40%) in the spectrum, indicating that CO loss is more favored than water loss. Both ions *m*/*z* 274 and *m*/*z* 264 lead to formation of ion with *m*/*z* 264 by a CO and water losses respectively. We also propose a dienone-phenol rearrangement, previous to water loss from ion *m*/*z* 264 to give the ion *m*/*z* 246. 

**Scheme 2:**
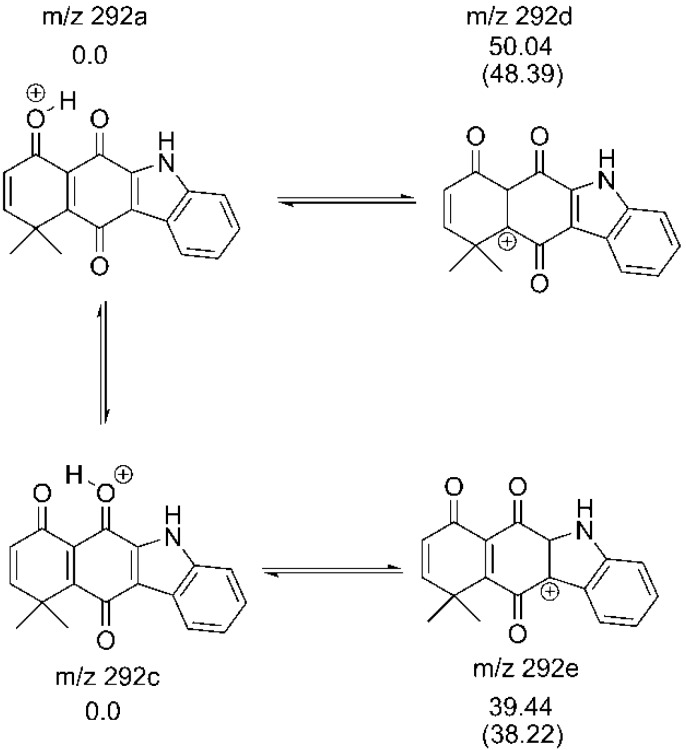
Proton transfer equilibria between O_1_ and O_2_ for **CQ1**.

**Scheme 3:**
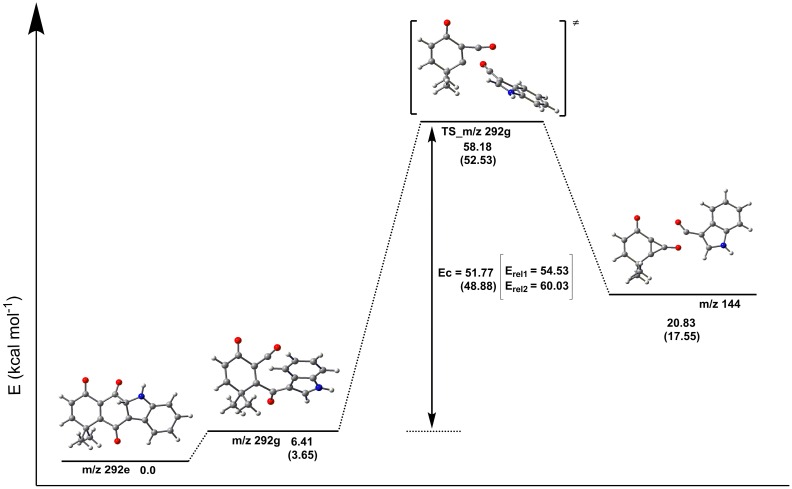
Energy profile of [CQ1 + H]^+^ fragmentation to give *m*/*z* 144. Energy relative 1 (*E*_rel1_) at M06-2x/6-311++G(3df,3pd) and Energy relative 2 (*E*_rel2_) at QCISD/6-31++G(d,p).

**Scheme 4:**
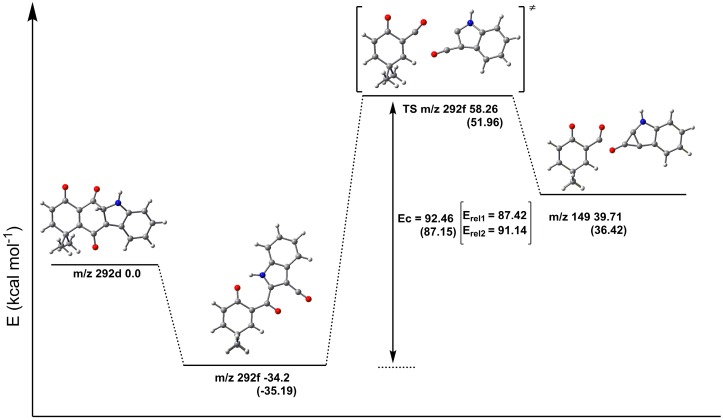
Energy profile of [CQ1 + H]^+^ fragmentation to *m*/*z* 149. Energy relative 1 (*E*_rel1_) at M06-2x/6-311++G(3df,3pd) and Energy relative 2 (*E*_rel2_) at QCISD/6-31++G(d,p).

For the fragments with *m*/*z* 144 (100%) and *m*/*z* 149 (40%), a parallel mechanism is plausible, starting from the proton transfer equilibrium between O_1_ and O_2_ (Scheme 2). Both PA and GB have the same values for protonation on O_1_ and O_2_ (Table 2). The ion *m*/*z* 292e is 10.57 and 10.17 kcal·mol^−1^ lower than 292d in enthalpy and Gibbs free energy, respectively. Scheme 3 and Scheme 4 show the DFT calculated energy profile for the *m*/*z* 292e and 292d ion fragmentation route. The critical energy Ec, defined as the barrier energy for the transition state are 51.77 kcal·mol^−1^ (Gibbs critical energy 48.88 kcal mol^−1^) for TS_*m*/*z* 292g (Scheme 3) and 92.46 kcal·mol^−1^ (Gibbs critical energy 87.15 kcal·mol^−1^) for TS_*m*/*z* 292f (Scheme 4), respectively. In order to obtain a most accurate description, relative energy for the TSs at DFT Minnesota functional M06-2x/6-311++G(3df,3pd) (*E*_rel1_) and ab-initio quadratic configuration interaction, with single and double excitation at QCISD/6-31++G(d,p) (*E*_rel2_) level were obtained at the B3LYP optimized geometries. M06-2x energies underrate the energies of TSs compared with high level calculation at QCISD. For TS_*m*/*z* 292g the difference is 5.50 kcal·mol^−1^, while for TS_*m*/*z* 292f it is 3.72 kcal·mol^−1^. These results are in agreement with the differences in the observed relative populations for ions with *m*/*z* 144(100) and *m*/*z* 149(40). 

A very similar fragmentation route was found for **CQ2**. Compared with **CQ1**, the only difference was an additional methyl radical loss from the *m*/*z* 278 ion, obtained initially by CO loss. 

**Scheme 5:**
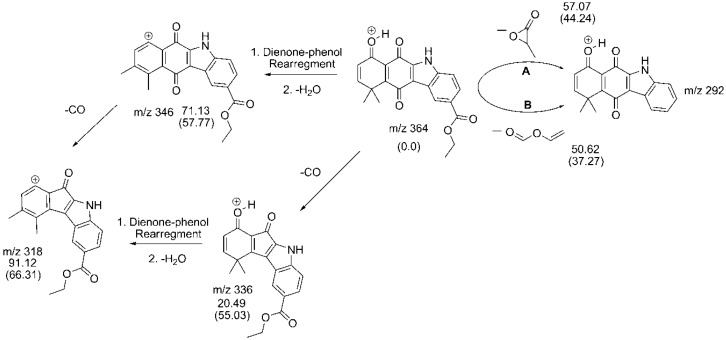
Fragmentation pattern for protonated **CQ3**.

Moreover, **CQ3** and **CQ4** display a fragmentation similar to **CQ1**, but the substituent present in the aromatic ring leads to some different fragmentation steps. Table 3 shows that CO and water loss are present in **CQ3**. Additionally, the loss of –C_3_H_4_O_2_ fragment was also observed. Scheme 5 shows the fragmentation route proposed for **CQ3**; we propose that water loss goes in a similar way than **CQ1** and **CQ2**, with a dienone-phenol rearrangement followed by water loss to give the fragment with *m*/*z* 346. Also, the CO loss proceeds in a similar way to **CQ1** and **CQ2**, to give the fragment *m*/*z* 336 with the lower enthalpy and free energy, according to the higher percentages observed for this ion. Finally, both routes lead to the same molecular ion with *m*/*z* 318, by CO loss from ion with *m*/*z* 346 and by water loss from ion with *m*/*z* 336. For loss of fragment C_3_H_4_O_2_ we investigate two possibilities, loss of 3-methyloxiran-2-one (**A**) and loss of vinyl formate (**B**). The formation of linear specie vinyl formate is thermodynamically favorable, as reflects their lower enthalpy and free energy. Ion with *m*/*z* 292 has the same structure than the protonated molecular ion of **CQ1** and experiences a similar subsequent fragmentation pathway (see Scheme 1). 

**Scheme 6:**
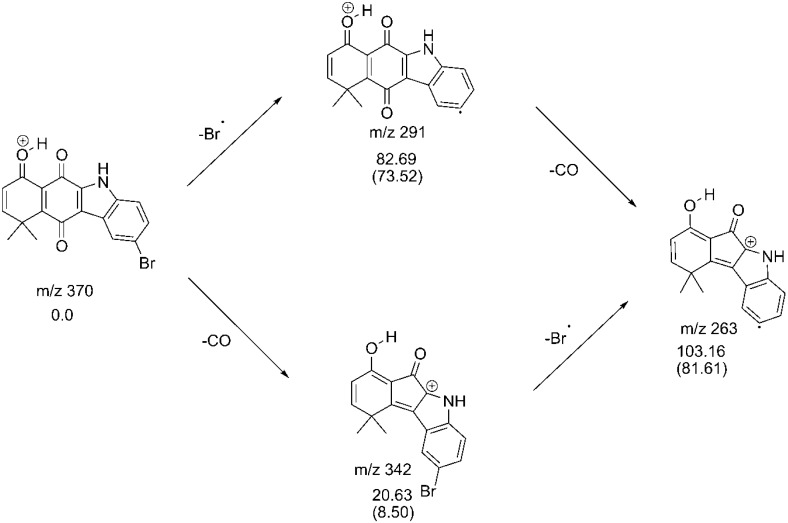
Fragmentation pattern for protonated **CQ4**.

Protonated molecular ion of **CQ4** presents a Br loss to give the fragment with *m*/*z* 291 and a CO loss to give a fragment with *m*/*z* 342 (Scheme 6). Enthalpies and free energies indicate that a fragment with *m*/*z* 342 should be more easily formed, but the spectrum shows a greater ratio for *m*/*z* 291. This fragment also corresponds to the protonated radical cation of **CQ1**. Both ions with *m*/*z* 291 and *m*/*z* 342 can lead to a fragment with *m*/*z* 263 by CO and radical Br losses, respectively.

## 3. Experimental

### 3.1. Mass Spectrometry 

Stock solutions for ESI-MS experiments were prepared by dissolving the compound of interest in 200 μL of acetonitrile. Working solutions were prepared in two different ways: (a) by mixing 20 μL of stock solution and 80 μL of acetonitrile or (b) by adding 60 μL of acetonitrile, 16 μL of water and 4 μL of formic acid 5% *v*/*v*, to 20 μL of stock solution. Spectra were acquired in an Esquire 4000 ESI-IT ion trap mass spectrometer (Bruker Daltonik GmbH, Bremen, Germany). Working solutions were analyzed by direct infusion (50 μL) at a flow rate of 3.0 μL/min using a syringe pump (Cole-Parmer, Vernon Hills, IL, USA). The ionization process was performed by electrospray at 4000 V, assisted by nitrogen as nebulizer gas at a temperature of 300 °C, pressure of 10 psi, and flow rate of 5 L/min. 

Collision-induced dissociation (CID) was performed by collisions with helium background gas present in the trap. Fragmentation was set with Smart Frag between 30% and 200%, with an isolation width of 4.0 *m*/*z*; 1.0 V fragmentation amplitude; fragmentation time of 40 ms; fragmentation delay of 0 ms, and the average of 5 spectra were obtained in all cases. Spectra can be found in Figures S1–S4 (Supplementary Materials).

### 3.2. Synthetic Methodology

Melting points were determined on a hot-stage apparatus and are uncorrected. The IR spectra were recorded on a FT-IR Bruker IFS 55 spectrophotometer (Bruker Daltonik GmbH, Bremen, Germany) from KBr discs. ^1^H and ^13^C NMR spectra were acquired using a Bruker AVANCE 400 spectrometer (Bruker Daltonik GmbH, Bremen, Germany) operating at 400 MHz (^1^H) or 100 MHz (^13^C). All measurements were carried out at a probe temperature of 300 K, in CDCl_3_ or DMSO-*d*_6_ containing tetramethylsilane (TMS) as an internal standard.

#### Synthesis of Carbazolequinones: General Procedure

In a Schlenk tube under inert atmosphere, a mixture of one equivalent of the respective anilinoquinone and one equivalent of Pd(OAc)_2_ in glacial acetic acid, is heated under reflux by 4 h. and then filtered. The filtered is extracted 3 times with ethyl acetate and washed with a solution of sodium bicarbonate; the organic phase is dried with anhydrous sodium sulfate and then evaporated under vacuum. Column chromatography on silica gel with hexane:EtOAc 2:1 mixture as eluent allow to obtain pure carbazolequinones.

10,10-Dimethyl-5*H*-benzo[b]carbazole-6,7,11(10*H*)-trione (**CQ1**)

Seventy milligrams (0.24 mmol) of 3-anilino-8,8-dimethylnaphthalene-1,4,5(8*H*)-trione and Pd(OAc)_2_ 54 mg (0.24 mmol) in glacial acetic acid (4 mL) yield 36 mg of **CQ1** (52%). ^1^H NMR (400 MHz, CDCl_3_) δ: 1.71 (s, 6H), 6.36 (d, *J* = 10 Hz, 1H), 6.81 (d, *J* = 10 Hz, 1H), 7.39 (t, *J* = 8 Hz, 1H), 7.46 (t, *J* = 8 Hz, 1H), 7.59 (d, *J* = 8 Hz, 1H), 8.27 (d, *J* = 8 Hz, 1H), 9.57 (s, 1H). ^13^C NMR (101 MHz, CDCl_3_) δ: 184.87, 183.64, 179.00, 160.50, 159.17, 158.84, 138.42, 135.57, 128.58, 125.65, 125.12, 124.25, 123.98, 118.57, 114.19, 40.69, 30.67. IR(KBr): 3237, 1683, 741 cm^−1^. M.p.: 297–299 °C.

4,10,10-Trimethyl-5*H*-benzo[b]carbazole-6,7,11(10*H*)-trione (**CQ2**)

Sixty-eight milligrams (0.22 mmol) of 8,8-dimethyl-3-[(2-methylphenyl)amino]naphthalene-1,4,5(8*H*)-trione and Pd(OAc)_2_ 49 mg (0.22 mmoles) yield 32 mg of carbazolequinone 7 (77%). ^1^H NMR (400 MHz, CDCl_3_) δ: 1.71 (s, 6H), 2.58 (s, 3H), 6.35 (d, *J* = 10 Hz, 1H), 6.80 (d, *J* = 10 Hz, 1H), 7.24 (d, *J* = 8 Hz, 1H), 7.30 (t, *J* = 8 Hz, 1H), 8.1 (d, *J* = 8 Hz, 1H), 9.43 (s, 1H); ^13^C NMR (101 MHz, CDCl_3_) δ 184.82, 183.73, 178.99, 160.60, 158.97, 138.12, 134.99, 130.96, 128.98, 128.27, 125.99, 124.79, 123.60, 121.63, 118.88, 40.68, 36.62, 27.72. IR: 3215, 1686, 811 cm^−1^. M.p.: 291–293 °C.

2-Bromo-10,10-dimethyl-5H-benzo[b]carbazole-6,7,11(10*H*)-trione (**CQ3**)

Forty-one milligrams of 3-[(4-bromophenyl)amino]-8,8-dimethylnaphthalene-1,4,5(8*H*)-trione (0.11 mmoles) and Pd(OAc)_2_ 25 mg (0.11 mmoles) yield 17 mg of **CQ3** (39%). ^1^H NMR (400 MHz, DMSO-*d*_6_) δ: 1.66 (s, 6H), 6.31 (d, *J* = 10 Hz, 1H), 7.06 (d, *J* = 10 Hz, 1H), 7.57 (d, *J* = 8 Hz, 1H), 7.62 (d, *J* = 8 Hz, 1H), 9.47 (s, 1H). ^13^C NMR (100 MHz, DMSO) δ 187.55, 181.64, 181.44, 178.41, 177.03, 159.62, 145.04, 144.27, 137.91, 130.74, 130.25, 128.01, 126.40, 118.03, 112.43, 31.24, 27.63. IR: 3220, 1690, 817 cm^−1^. M.p.: 272–274 °C.

Ethyl10,10-dimethyl-6,7,11-trioxo-6,7,10,11-tetrahydro-5*H*-benzo[b]carbazole-2 carboxylate (**CQ4**)

Ninety-three miligrams (0.25 mmol) of ethyl 4-[(5,5-dimethyl-1,4,8-trioxo-1,4,5,8-tetrahydronaphthalen-2-yl)amino]benzoate and Pd(OAc)_2_ 57 mg (0.25 mmol) yield 21 mg of **CQ4** (23%). ^1^H NMR (400 MHz, DMSO-*d*_6_) δ: 1.36 (t, *J* = 7 Hz, 3H), 1.61 (s, 6H), 4.35 (q, *J* = 7 Hz, 2H), 6.26 (d, *J* = 10 Hz, 1H), 7.02 (d, *J* = 10 Hz, 1H), 7.63 (d, *J* = 9 Hz, 1H), 7.98 (d, *J* = 9 Hz, 1H), 8.72 (s, 1H), 13.19 (s, 1H). ^13^C NMR (101 MHz, DMSO-*d*_6_) δ: 184.31, 183.88, 178.88, 174.23, 167.23, 159.83, 159.21, 141.58, 137.86, 131.20, 128.07, 127.00, 125.80, 124.46, 118.38, 115.20, 61.75, 30.52, 27.77, 15.46. IR: 3263, 1687, 1278. IR: 3263, 1687, 1278 cm^−1^. M.p.: 265–267 °C.

### 3.3. Computational Methodology 

The calculations were carried out using the Gaussian 09 [64] program package, running in a Microsystem cluster of blades. No symmetry constraints were imposed on the optimizations, which were performed at DFT B3LYP/6-31G(d,p) level. No imaginary vibrational frequencies were found at the optimized geometries, indicating that they are true minima of the potential energy surface.

Protonation sites were obtained on the basis of molecular electrostatic potential (MEP) [65], local Fukui indexes [66], Parr functions [58], proton affinity (PA) and gas-phase basicities (GB) among several protonated forms. GB was calculated from the Gibbs free energies of the following reaction in gas phase: CQ + H^+^ → CQH^+^, where a Gibbs energy of 6.28 kcal·mol^−1^ was considered for the proton [67].

The energies for the products and the corresponding transition state (TS) were obtained relative to their respective precursors. Relative energies for TS were obtained at DFT M06-2x/6-311++G(3df,3pd)//B3LYP/6-31G(d,p) and ab-initio QCISD/6-31++G(d,p)//B3LYP/6-31G(d,p) levels. Only one imaginary vibrational frequency was found for the TS. Intrinsic reaction coordinate (IRC) calculations were carried out to verify the connections of the transition states with reactants and products [68]. Cartesian coordinates and energies of optimized structures can be found in Supplementary Materials (Tables S1–S82).

## 4. Conclusions

A series of four new *o*-carbonyl carbazolquinones were synthetized from anilinquinones previously prepared. The influence of the substituent in ring D on the fragmentation route obtained by ESI-MS/MS analysis was computationally assisted and allowed a detailed interpretation of the experimental results. The most favorable protonation site for all molecules was established through molecular electrostatic potential, local Fukui functions and local Parr function for electrophilic attack, as well as proton affinity and gas phase basicity. On the basis of this initial analysis, fragmentation routes were proposed and supported by theoretical calculations.

The unusual kind of fragmentations leading to *m*/*z* 144 and *m*/*z* 149 ions for **CQ1** were studied in detail. The critical energies for the transition states for the proposed fragmentations were in agreement with the differences in the observed relative populations for both ions, supporting the proposed mechanism. 

These results have importance in establishing a guide for future analysis of this kind of carbazolequinones and similar scaffolds, with possible applications in drug development and organic materials. 

## Figures and Tables

**Table:** 

*R*	CQ	Yield (%)
–H	**1**	52
1′–CH_3_	**2**	77
3′–CO^(5′)^O^(6′)^Et	**3**	23
3′–Br	**4**	39

**Table 1:** Fukui function (***f***^−^) and Parr function (***P***^−^) for electrophilic attack.

Atom	Fukui Function for Electrophilic Attack				Parr Function for Electrophilic Attack			
	CQ1	CQ2	CQ3	CQ4	CQ1	CQ2	CQ3	CQ4
O1	0.149	0.111	0.148	0.149	0.262	0.224	0.270	0.282
O2	0.091	0.067	0.089	0.087	0.061	0.043	0.066	0.080
O3	0.054	0.054	0.052	0.049	0.054	0.050	0.053	0.038
N4	0.008	0.012	−0.008	0.006	0.110	0.122	0.108	0.103
O5′			0.026				−0.003	
O6′			−0.001				0.009	

**Table 2:** Calculated Proton Affinities (PA) and Gas-Phase Basicity (GB), in kcal·mol^−1^, for the protonation of oxygens 1 and 2 (same value for both oxygens) for all CQs. Results for all protonation sites are in the Supplementary Materials.

Compound	PA	GB
**CQ1**	240.0	231.3
**CQ2**	241.0	232.4
**CQ3**	238.4	229.9
**CQ4**	238.1	229.5

**Table 3:** Ionic species observed in the electrospray ionization mass spectrometry (ESI-MS) spectra of CQs.

Ion	CQ1 *m*/*z*	CQ2 *m*/*z*	CQ3 *m*/*z*	CQ4 *m*/*z*
[M + H]^+^	292	306	364	370
[M + Na]^+^	314	328	386	^a^
[M + K]^+^	330	344	^a^	^a^

^a^ Not observed.

**Table 4:** Molecular and main fragment ^ions^ observed by ESI-MS^n^ analysis of **CQ1** to **CQ4**.

Compound	[M + H]^+^ *m*/*z* (%)	MS^2^ *m*/*z* (%)	MS^3^ *m*/*z* (%)
**CQ1**	292	274(40) –H_2_O	246(100) –CO
		264(100) –CO	246(86) –H_2_O
		149(40) –C_9_H_5_NO	121(100) –CO
		144(100) –C_9_H_8_O_2_	116(100) –CO
**CQ2**	306	288(48) –H_2_O	260(100) –CO
		278(100) –CO	263(100) –CH_3_ 260(95) –H_2_O
		158(61)	130(100) –CO
		149(13)	121(100) –CO
**CQ3**	364	346(15) –H_2_O	318(100) –CO
		336(50) –CO	318(35) –H_2_O
		292(100) –C_3_H_4_O_2_	264(100) –CO 149(35) –C_9_H_9_O_2_ 144(100) –C_9_H_6_NO
**CQ4**	370	291(100) –Br	263(100) –CO
		342(10) –CO	263(100) –Br

## Supplementary Materials

Supplementary materials can be found at http://www.mdpi.com/1422-0067/17/7/1071/s1.
