# Supplementary material for: Protonation Sites, Tandem Mass Spectrometry and Computational Calculations of o-Carbonyl Carbazolequinone Derivatives
Source: Int J Mol Sci. 2016 Jul 5;17(7):1071. doi: 10.3390/ijms17071071 (PMC4964447; doi:10.3390/ijms17071071)
Supplement: Supplementary file 1 [file ijms-17-01071-s001.pdf]

Supplementary Materials: Protonation Sites, Tandem Mass Spectrometry and Computational Calculations of *o*-Carbonyl Carbazolequinone Derivatives

Maximiliano Martínez-Cifuentes, Graciela Clavijo-Allancan, Pamela Zuñiga-Hormazabal, Braulio Aranda, Andrés Barriga, Boris Weiss-López and Ramiro Araya-Maturana

1. Optimized Geometries and Energies

DFT B3LYP/6-31G(d,p) level

1.1. Neutral Species

Table S1. Cartesian coordinates for **CQ1**.

C	−4.091623	0.700303	0.060087
C	−3.959467	−0.624404	0.152651
C	−2.927999	1.564045	−0.175800
C	−1.583404	0.914506	0.003062
C	−1.454016	−0.442176	0.025377
C	−2.660131	−1.375078	0.066322
C	−2.730824	−2.253784	−1.219336
C	−2.585069	−2.292191	1.324096
C	−0.390424	1.826709	0.146383
C	−0.071276	−1.099490	−0.019233
C	0.904159	1.155932	0.038883
C	1.074480	−0.209948	−0.030532
N	2.116335	1.776309	0.076010
C	2.490769	−0.459243	−0.047211
C	3.115075	0.818670	0.018617
C	3.288241	−1.612202	−0.107050
C	4.503921	0.968540	0.019325
C	4.666215	−1.462819	−0.103798
C	5.266221	−0.188480	−0.041793
O	−3.050775	2.716806	−0.547961
O	−0.462438	3.015301	0.398271
O	0.050939	−2.316993	−0.064045
H	−2.783011	−1.627626	−2.113145
H	−3.632004	−2.872105	−1.186756
H	−1.861784	−2.905131	−1.286370
H	−2.527352	−1.694294	2.237066
H	−1.722641	−2.952589	1.273211
H	−3.491171	−2.901650	1.377177
H	2.230811	2.777115	0.144431
H	−5.057268	1.192651	0.081090
H	−4.841116	−1.250089	0.270026
H	6.347331	−0.111392	−0.041148
H	2.825038	−2.589017	−0.155405
H	5.299770	−2.340903	−0.150684
H	4.968157	1.946614	0.068028

Table S2. Energies for **CQ1**.

Zero-point correction	0.267187 (Hartree/Particle)
Thermal correction to Energy	0.284824
Thermal correction to Enthalpy	0.285769
Thermal correction to Gibbs Free Energy	0.222144
Sum of electronic and zero-point Energies	−974.146119
Sum of electronic ad thermal Energies	−974.128481
Sum of electronic and thermal Enthalpies	−974.127537
Sum of electronic and thermal Free Energies	−974.191161

Table S3. Cartesian coordinates for **CQ2**.

C	−4.274958	0.994172	0.067551
C	−4.272262	−0.336932	0.161872
C	−3.033558	1.740238	−0.173511
C	−1.758110	0.963013	0.001975
C	−1.761533	−0.399891	0.027350
C	−3.052765	−1.210795	0.072138
C	−3.213024	−2.077493	−1.213606
C	−3.064962	−2.132137	1.329177
C	−0.480458	1.754398	0.136702
C	−0.449821	−1.189801	−0.019824
C	0.740836	0.959335	0.028716
C	0.777624	−0.417042	−0.034764
N	2.008187	1.457618	0.059464
C	2.162408	−0.804263	−0.053286
C	2.909201	0.405331	0.004168
C	2.840595	−2.031571	−0.107878
C	4.312098	0.442081	0.001386
C	4.224975	−2.008540	−0.107332
C	4.942281	−0.795446	−0.054582
O	−3.045469	2.899176	−0.546764
O	−0.434876	2.946233	0.380825
O	−0.449039	−2.413352	−0.064963
H	−3.203181	−1.449061	−2.107260
H	−4.171640	−2.602255	−1.179794
H	−2.413476	−2.812341	−1.281711
H	−2.949932	−1.543381	2.242678
H	−2.269446	−2.871620	1.276893
H	−4.025010	−2.652572	1.382450
H	2.217677	2.443029	0.122075
H	−5.188026	1.578269	0.090863
H	−5.210329	−0.873659	0.282508
C	5.067483	1.743603	0.057441
H	6.026819	−0.827243	−0.056775
H	2.286085	−2.959621	−0.149858
H	4.776727	−2.940598	−0.149704
H	4.833167	2.307517	0.967199
H	4.827831	2.385538	−0.797483
H	6.144202	1.568672	0.046728

Table S4. Energies for **CQ2**.

Zero-point correction	0.294619 (Hartree/Particle)
Thermal correction to Energy	0.314038
Thermal correction to Enthalpy	0.314982
Thermal correction to Gibbs Free Energy	0.247478
Sum of electronic and zero-point Energies	−1013.447303
Sum of electronic and thermal Energies	−1013.427885
Sum of electronic and thermal Enthalpies	−1013.426941
Sum of electronic and thermal Free Energies	−1013.494444

Table S5. Cartesian coordinates for **CQ3**.

C	−5.379772	0.746743	−0.001142
C	−4.712933	1.895332	0.129597
C	−4.673676	−0.518903	−0.234900
C	−3.184803	−0.487726	−0.020592
C	−2.506139	0.692655	0.042011
C	−3.217438	2.040752	0.092582
C	−2.808097	2.813449	1.383044
C	−2.878695	2.900268	−1.162821
C	−2.479300	−1.814698	0.108660
C	−0.975709	0.718639	0.036069
C	−1.020560	−1.738511	0.029371
C	−0.299835	−0.566326	0.004322
N	−0.173374	−2.808032	0.056440
C	1.093148	−0.927401	0.006267
C	1.130017	−2.350557	0.036292
C	2.292154	−0.205648	−0.011944
C	2.334103	−3.061595	0.041377
C	3.491864	−0.911509	−0.004375
C	3.504456	−2.326322	0.021903
O	−5.253537	−1.512930	−0.631953
O	−3.039505	−2.872464	0.326801
O	−0.358001	1.774792	0.036386
H	−3.030805	2.222943	2.275206
H	−3.382613	3.741659	1.441215
H	−1.748716	3.058869	1.370477
H	−3.156862	2.373753	−2.078829
H	−1.816926	3.136263	−1.191580
H	−3.445030	3.834785	−1.125390
H	−0.484562	−3.768005	0.095278
H	−6.462798	0.699924	−0.012756
H	−5.258883	2.828602	0.245516
H	2.352911	−4.144843	0.061864
H	4.465713	−2.824249	0.026961
H	2.282184	0.874602	−0.031871
C	4.813263	−0.219109	−0.023715
O	4.693633	1.122891	−0.054131
C	5.928274	1.881932	−0.074820
H	6.506697	1.580612	−0.951077
H	6.509301	1.624364	0.813602
C	5.561175	3.351020	−0.110723
H	6.471876	3.955735	−0.127798

H	4.979610	3.632167	0.770050
H	4.975980	3.587784	−1.002081
O	5.880855	−0.791495	−0.013683

Table S6. Energies for **CQ3**.

Zero-point correction	0.337803 (Hartree/Particle)
Thermal correction to Energy	0.361476
Thermal correction to Enthalpy	0.362420
Thermal correction to Gibbs Free Energy	0.283677
Sum of electronic and zero-point Energies	−1241.351749
Sum of electronic and thermal Energies	−1241.328076
Sum of electronic and thermal Enthalpies	−1241.327131
Sum of electronic and thermal Free Energies	−1241.405874

Table S7. Cartesian coordinates for **CQ4**.

C	−5.295181	−0.228925	0.024620
C	−4.800012	−1.462821	0.140363
C	−4.415786	0.921894	−0.214005
C	−2.945276	0.674989	−0.013259
C	−2.443401	−0.591809	0.031864
C	−3.342107	−1.823193	0.077466
C	−3.149666	−2.703134	−1.194941
C	−3.029178	−2.666189	1.350263
C	−2.056830	1.885475	0.125463
C	−0.932838	−0.838526	0.009579
C	−0.623749	1.600966	0.042351
C	−0.080372	0.335699	−0.003008
N	0.366813	2.534997	0.081038
C	1.348721	0.491171	−0.001403
C	1.592011	1.892116	0.049622
C	2.428711	−0.403739	−0.033730
C	2.886043	2.416459	0.061934
C	3.703308	0.133548	−0.018015
C	3.943210	1.520536	0.028390
O	−4.848602	1.990742	−0.604098
O	−2.458203	3.010912	0.354750
O	−0.472798	−1.972342	−0.018215
H	−3.356730	−2.125797	−2.099079
H	−3.848219	−3.543569	−1.163376
H	−2.135066	−3.093522	−1.241696
H	−3.154529	−2.065167	2.254344
H	−2.015116	−3.057477	1.317541
H	−3.727116	−3.505961	1.404693
H	0.197309	3.529088	0.133956
H	−6.360032	−0.025625	0.028768
H	−5.473868	−2.307870	0.259848
H	3.067871	3.484046	0.098620
H	4.961731	1.885525	0.038495
H	2.258015	−1.470430	−0.070089
Br	5.215121	−1.045995	−0.062003

Table S8. Energies for **CQ4**.

Zero-point correction	0.256854 (Hartree/Particle)
Thermal correction to Energy	0.276067
Thermal correction to Enthalpy	0.277011
Thermal correction to Gibbs Free Energy	0.208568
Sum of electronic and zero-point Energies	−3547.697444
Sum of electronic and thermal Energies	−3547.678231
Sum of electronic and thermal Enthalpies	−3547.677286
Sum of electronic and thermal Free Energies	−3547.745730

1.2. Protonated Species for CQ1

Table S9. Cartesian coordinates for **CQ1_O1**.

C	4.057977	0.674236	−0.000007
C	3.932355	−0.672435	0.000082
C	2.877679	1.473681	−0.000050
C	1.559621	0.857820	−0.000031
C	1.434717	−0.509823	0.000120
C	2.639516	−1.415264	0.000188
C	2.638228	−2.329162	1.280038
C	2.638120	−2.329377	−1.279518
C	0.380261	1.785872	−0.000291
C	0.036096	−1.142421	0.000167
C	−0.902213	1.158036	−0.000104
C	−1.086572	−0.224121	0.000070
N	−2.118448	1.781876	−0.000123
C	−2.495078	−0.461097	0.000006
C	−3.114421	0.829974	−0.000083
C	−3.297283	−1.617801	0.000047
C	−4.507192	0.989880	−0.000128
C	−4.671065	−1.454891	0.000005
C	−5.266898	−0.167229	−0.000081
O	3.003530	2.758192	−0.000032
O	0.545002	3.023957	−0.000398
O	−0.092665	−2.362255	0.000016
H	2.646576	−1.728376	2.192773
H	3.533413	−2.955778	1.268763
H	1.756240	−2.967133	1.269316
H	2.646450	−1.728745	−2.192354
H	1.756094	−2.967296	−1.268633
H	3.533269	−2.956044	−1.268190
H	−2.244570	2.785325	−0.000216
H	5.016734	1.180680	−0.000023
H	4.827057	−1.290376	0.000126
H	−6.349111	−0.089667	−0.000107
H	−2.838940	−2.599325	0.000128
H	−5.314379	−2.328386	0.000049
H	−4.967569	1.971995	−0.000196
H	2.057855	3.165592	0.000149

Table S10. Energies for **CQ1_O1**.

Zero-point correction	0.281366 (Hartree/Particle)
Thermal correction to Energy	0.298670
Thermal correction to Enthalpy	0.299614
Thermal correction to Gibbs Free Energy	0.237298
Sum of electronic and zero-point Energies	−974.290473
Sum of electronic and thermal Energies	−974.273169
Sum of electronic and thermal Enthalpies	−974.272225
Sum of electronic and thermal Free Energies	−974.334541

Table S11. Cartesian coordinates for **CQ1_O2**.

C	−4.058036	0.674349	−0.000194
C	−3.932514	−0.672358	−0.000298
C	−2.877667	1.473760	−0.000028
C	−1.559581	0.857801	0.000045
C	−1.434791	−0.509797	−0.000003
C	−2.639663	−1.415286	−0.000066
C	−2.638365	−2.329701	−1.279427
C	−2.638609	−2.328720	1.280156
C	−0.380223	1.785952	0.000034
C	−0.035870	−1.142710	0.000141
C	0.902214	1.157952	−0.000090
C	1.086605	−0.224150	−0.000046
N	2.118461	1.781829	−0.000267
C	2.495119	−0.461149	−0.000036
C	3.114487	0.829925	−0.000186
C	3.297362	−1.617846	0.000031
C	4.507255	0.989923	−0.000286
C	4.671162	−1.454881	−0.000056
C	5.266996	−0.167204	−0.000211
O	−3.003525	2.758269	−0.000027
O	−0.544806	3.024082	0.000045
O	0.092924	−2.362478	0.000463
H	−2.646292	−1.729350	−2.192476
H	−3.533787	−2.956023	−1.268115
H	−1.756635	−2.968046	−1.268274
H	−2.646733	−1.727622	2.192707
H	−1.756819	−2.967001	1.269578
H	−3.533995	−2.955089	1.269115
H	2.244615	2.785273	−0.000055
H	−5.016789	1.180838	−0.000291
H	−4.827315	−1.290194	−0.000510
H	6.349227	−0.089618	−0.000284
H	2.839070	−2.599416	0.000139
H	5.314476	−2.328403	−0.000016
H	4.967588	1.972078	−0.000410
H	−2.057866	3.165621	0.000039

Table S12. Energies for **CQ1_O2**.

Zero-point correction	0.281361 (Hartree/Particle)
Thermal correction to Energy	0.298664
Thermal correction to Enthalpy	0.299608
Thermal correction to Gibbs Free Energy	0.237299
Sum of electronic and zero-point Energies	−974.290478
Sum of electronic and thermal Energies	−974.273175
Sum of electronic and thermal Enthalpies	−974.272231
Sum of electronic and thermal Free Energies	−974.334540

Table S13. Cartesian coordinates for **CQ1_O3**.

C	−4.108561	0.710348	0.008778
C	−3.965030	−0.617469	0.089374
C	−2.950372	1.590246	−0.154998
C	−1.599398	0.930265	0.005332
C	−1.455406	−0.433084	0.023700
C	−2.663316	−1.376512	0.049278
C	−2.715589	−2.269797	−1.229552
C	−2.636684	−2.266624	1.331692
C	−0.412643	1.845725	0.125658
C	−0.083988	−1.012438	0.005778
C	0.897776	1.168193	0.028546
C	1.066182	−0.224313	−0.026765
N	2.087713	1.767531	0.061418
C	2.504476	−0.470947	−0.044331
C	3.109275	0.814811	0.013931
C	3.332724	−1.596902	−0.103132
C	4.489008	1.009969	0.016196
C	4.715131	−1.412905	−0.098514
C	5.285455	−0.129939	−0.038950
O	−3.042497	2.771721	−0.439050
O	−0.455057	3.041009	0.348226
O	−0.009488	−2.330200	−0.002142
H	−2.736677	−1.655460	−2.133579
H	−3.630973	−2.867353	−1.208814
H	−1.870397	−2.955904	−1.281143
H	−2.600383	−1.650711	2.234393
H	−1.794691	−2.957578	1.334876
H	−3.554850	−2.859098	1.367649
H	2.196463	2.775731	0.119531
H	−5.080955	1.191511	0.008011
H	−4.846416	−1.250166	0.167619
H	6.364919	−0.026532	−0.037762
H	2.944172	−2.610737	−0.161272
H	5.365092	−2.279969	−0.144917
H	4.923061	2.003142	0.059941
H	0.909749	−2.641588	−0.022872

Table S14. Energies for **CQ1_O3**.

Zero-point correction	0.281072 (Hartree/Particle)
Thermal correction to Energy	0.299022
Thermal correction to Enthalpy	0.299967
Thermal correction to Gibbs Free Energy	0.235403
Sum of electronic and zero-point Energies	−974.250033
Sum of electronic and thermal Energies	−974.232083
Sum of electronic and thermal Enthalpies	−974.231139
Sum of electronic and thermal Free Energies	−974.295702

Table S15. Cartesian coordinates for **CQ1_N4**.

C	−4.130669	0.685629	0.031946
C	−3.991171	−0.644907	0.089514
C	−2.972503	1.570851	−0.107969
C	−1.619711	0.902650	0.010343
C	−1.486332	−0.460150	0.016646
C	−2.688675	−1.397268	0.040911
C	−2.721145	−2.288116	−1.240126
C	−2.635434	−2.304123	1.310277
C	−0.432000	1.814150	0.102265
C	−0.116888	−1.104124	−0.021208
C	0.849486	1.132450	−0.068452
C	1.063623	−0.190563	−0.063452
N	2.139043	1.861516	−0.018301
C	2.496209	−0.465812	−0.044928
C	3.162193	0.771470	−0.012122
C	3.245240	−1.642520	−0.047928
C	4.532687	0.913844	0.007281
C	4.638675	−1.528734	−0.023602
C	5.271359	−0.280497	0.003164
O	−3.067736	2.764415	−0.339658
O	−0.432514	3.008825	0.358711
O	0.073131	−2.309504	−0.033804
H	−2.758243	−1.673663	−2.143870
H	−3.619728	−2.910960	−1.219620
H	−1.851793	−2.944426	−1.282749
H	−2.608594	−1.701260	2.222329
H	−1.769611	−2.965195	1.287376
H	−3.537148	−2.921906	1.342404
H	2.165133	2.466942	0.815423
H	−5.101743	1.169588	0.040661
H	−4.874481	−1.276530	0.158146
H	6.354453	−0.227613	0.022984
H	2.750273	−2.605767	−0.070429
H	5.244701	−2.428368	−0.026216
H	5.026329	1.879867	0.030203
H	2.234429	2.493403	−0.825882

Table S16. Energies for **CQ1_N4**.

Zero-point correction	0.281463 (Hartree/Particle)
Thermal correction to Energy	0.299421
Thermal correction to Enthalpy	0.300365
Thermal correction to Gibbs Free Energy	0.235816
Sum of electronic and zero-point Energies	−974.217276
Sum of electronic and thermal Energies	−974.199318
Sum of electronic and thermal Enthalpies	−974.198373
Sum of electronic and thermal Free Energies	−974.262923

1.3. Protonated Species for CQ2

Table S17. Cartesian coordinates for **CQ2_O1**.

C	4.238253	0.980132	−0.000136
C	4.249981	−0.372236	0.000023
C	2.982700	1.655617	−0.000206
C	1.734407	0.909301	−0.000071
C	1.748513	−0.464310	0.000034
C	3.039221	−1.242765	0.000085
C	3.130920	−2.152245	1.279639
C	3.130893	−2.152335	−1.279428
C	0.467119	1.712794	−0.000020
C	0.422160	−1.235917	0.000094
C	−0.743456	0.957881	0.000018
C	−0.787460	−0.436287	0.000058
N	−2.017304	1.453548	−0.000011
C	−2.164192	−0.815497	0.000075
C	−2.912100	0.405419	0.000024
C	−2.842522	−2.048040	0.000130
C	−4.321410	0.448620	0.000026
C	−4.224748	−2.015060	0.000124
C	−4.943361	−0.791920	0.000073
O	2.976490	2.946303	−0.000526
O	0.505888	2.961938	0.000003
O	0.418801	−2.462781	0.000140
H	3.078088	−1.554015	2.192570
H	4.085110	−2.684741	1.268292
H	2.317858	−2.875947	1.268257
H	3.078044	−1.554163	−2.192395
H	2.317824	−2.876030	−1.267973
H	4.085079	−2.684834	−1.268061
H	−2.244419	2.438722	−0.000056
H	5.140685	1.581215	−0.000234
H	5.202708	−0.896314	0.000078
C	−5.073895	1.752765	−0.000023
H	−6.028692	−0.828187	0.000072
H	−2.289628	−2.979303	0.000169
H	−4.783713	−2.944954	0.000159
H	−4.837167	2.355527	−0.885168
H	−4.837173	2.355589	0.885082
H	−6.151542	1.579680	−0.000021
H	1.992499	3.253717	−0.000754

Table S18. Energies for **CQ2_O1**.

Zero-point correction	0.308946 (Hartree/Particle)
Thermal correction to Energy	0.328025
Thermal correction to Enthalpy	0.328969
Thermal correction to Gibbs Free Energy	0.262808
Sum of electronic and zero-point Energies	−1013.585942
Sum of electronic and thermal Energies	−1013.566864
Sum of electronic and thermal Enthalpies	−1013.565919
Sum of electronic and thermal Free Energies	−1013.632081

Table S19. Cartesian coordinates for **CQ2_O2**.

C	−1.732241	0.898925	−0.000026
C	−1.758593	−0.466098	−0.000008
C	−3.057753	−1.233798	0.000047
C	−3.159034	−2.135227	−1.277265
C	−3.159095	−2.133730	1.278631
C	−0.465462	1.660813	0.000022
C	−0.436651	−1.242944	−0.000043
C	0.742144	0.952185	0.000056
C	0.788378	−0.444239	0.000026
N	2.023984	1.449863	0.000118
C	2.152941	−0.822367	−0.000043
C	2.907828	0.402293	0.000101
C	2.830770	−2.059083	−0.000266
C	4.316989	0.438276	0.000040
C	4.205903	−2.027432	−0.000319
C	4.929063	−0.801809	−0.000162
O	−2.904810	2.951583	−0.000051
O	−0.488340	2.946852	0.000032
O	−0.422360	−2.459292	0.000011
H	−3.086735	−1.537197	−2.187987
H	−4.126623	−2.640775	−1.276814
H	−2.372436	−2.886155	−1.272201
H	−3.086682	−1.534544	2.188558
H	−2.372557	−2.884722	1.274292
H	−4.126740	−2.639155	1.278696
H	2.262693	2.431909	0.000557
H	−5.138352	1.607239	−0.001216
H	−5.212901	−0.853016	−0.000579
C	5.074153	1.738056	0.000187
H	6.012625	−0.845631	−0.000208
H	2.278254	−2.988548	−0.000345
H	4.764860	−2.955353	−0.000478
H	4.840156	2.339280	0.885217
H	4.840140	2.339482	−0.884706
H	6.149414	1.560213	0.000157
H	−1.517589	3.223497	−0.000156

Table S20. Energies for **CQ2_O2**.

Zero-point correction	0.308944 (Hartree/Particle)
Thermal correction to Energy	0.328018
Thermal correction to Enthalpy	0.328963
Thermal correction to Gibbs Free Energy	0.262822
Sum of electronic and zero-point Energies	−1013.585944
Sum of electronic and thermal Energies	−1013.566870
Sum of electronic and thermal Enthalpies	−1013.565926
Sum of electronic and thermal Free Energies	−1013.632067

Table S21. Cartesian coordinates for **CQ2_O3**.

C	−4.285091	1.010815	0.010261
C	−4.274058	−0.324520	0.091467
C	−3.045377	1.772008	−0.152317
C	−1.766178	0.981622	0.004740
C	−1.758327	−0.389677	0.025424
C	−3.053826	−1.208732	0.053326
C	−3.195507	−2.094897	−1.223552
C	−3.116250	−2.094158	1.337901
C	−0.493249	1.774400	0.116733
C	−0.452118	−1.103768	0.006758
C	0.740654	0.968188	0.020162
C	0.770098	−0.434440	−0.029806
N	1.985162	1.444070	0.047016
C	2.176530	−0.823379	−0.049506
C	2.908167	0.395123	0.001216
C	2.883259	−2.028091	−0.104240
C	4.307751	0.475944	−0.000089
C	4.276536	−1.974899	−0.102917
C	4.967674	−0.754075	−0.051491
O	−3.022155	2.957673	−0.433408
O	−0.414510	2.970046	0.330215
O	−0.510301	−2.422978	0.002422
H	−3.156461	−1.483028	−2.128685
H	−4.165735	−2.598486	−1.200847
H	−2.422255	−2.861186	−1.274712
H	−3.024011	−1.481852	2.239091
H	−2.343600	−2.861785	1.345678
H	−4.086856	−2.596316	1.372420
H	2.192828	2.436438	0.100527
H	−5.204965	1.586090	0.008641
H	−5.213871	−0.866758	0.169164
C	5.034384	1.793581	0.050784
H	6.052937	−0.763876	−0.053379
H	2.397298	−2.998884	−0.156819
H	4.842867	−2.899081	−0.145837
H	4.784628	2.357390	0.957237
H	4.788268	2.422514	−0.812776
H	6.114718	1.640307	0.047517
H	0.373369	−2.824173	−0.019302

Table S22. Energies for **CQ2_O3**.

Zero-point correction	0.308505 (Hartree/Particle)
Thermal correction to Energy	0.328314
Thermal correction to Enthalpy	0.329259
Thermal correction to Gibbs Free Energy	0.260427
Sum of electronic and zero-point Energies	−1013.546671
Sum of electronic and thermal Energies	−1013.526862
Sum of electronic and thermal Enthalpies	−1013.525917
Sum of electronic and thermal Free Energies	−1013.594749

Table S23. Cartesian coordinates for **CQ2_N4**.

C	−4.319194	0.978187	0.037524
C	−4.307219	−0.359515	0.095400
C	−3.082112	1.748822	−0.105881
C	−1.799018	0.955159	0.010745
C	−1.796404	−0.414231	0.018275
C	−3.082550	−1.232588	0.043144
C	−3.202532	−2.112408	−1.240371
C	−3.114838	−2.144333	1.309694
C	−0.528645	1.748747	0.098094
C	−0.494837	−1.186779	−0.020895
C	0.680881	0.946769	−0.068826
C	0.767765	−0.391036	−0.062276
N	2.032826	1.547976	−0.022921
C	2.166626	−0.802418	−0.045390
C	2.948912	0.365461	−0.016422
C	2.796332	−2.045717	−0.047367
C	4.332518	0.402771	−0.000527
C	4.194052	−2.055837	−0.025990
C	4.936627	−0.871140	−0.003754
O	−3.064866	2.945797	−0.339283
O	−0.415046	2.939894	0.347112
O	−0.423416	−2.404880	−0.036125
H	−3.181158	−1.494482	−2.142257
H	−4.157088	−2.645531	−1.220206
H	−2.400298	−2.849023	−1.285918
H	−3.031226	−1.549477	2.223595
H	−2.314738	−2.883430	1.284185
H	−4.070596	−2.674850	1.340543
H	2.119880	2.151642	0.808076
H	−5.239617	1.552682	0.048047
H	−5.246590	−0.904060	0.165923
C	5.127953	1.682722	0.023066
H	6.020656	−0.925337	0.013551
H	2.214046	−2.958586	−0.066977
H	4.719171	−3.005084	−0.027475
H	4.901895	2.286737	0.909964
H	4.931059	2.300470	−0.861511
H	6.198277	1.472040	0.038523
H	2.187272	2.168157	−0.830693

Table S24. Cartesian coordinates for **CQ2_N4**.

Zero-point correction	0.308948 (Hartree/Particle)
Thermal correction to Energy	0.328770
Thermal correction to Enthalpy	0.329714
Thermal correction to Gibbs Free Energy	0.260831
Sum of electronic and zero-point Energies	−1013.514191
Sum of electronic and thermal Energies	−1013.494370
Sum of electronic and thermal Enthalpies	−1013.493425
Sum of electronic and thermal Free Energies	−1013.562308

1.4. Protonated Species for CQ3

Table S25. Cartesian coordinates for **CQ3_O1**.

C	5.338090	0.774345	0.000083
C	4.663849	1.947010	0.000103
C	4.597140	−0.443440	0.000030
C	3.142119	−0.431066	0.000009
C	2.460284	0.760817	0.000019
C	3.179127	2.085415	0.000056
C	2.799006	2.916132	−1.280053
C	2.798901	2.916116	1.280139
C	2.454337	−1.764911	0.000001
C	0.925138	0.755426	0.000037
C	1.026322	−1.726351	−0.000035
C	0.284447	−0.546861	−0.000014
N	0.178942	−2.800382	−0.000103
C	−1.095526	−0.917869	−0.000037
C	−1.121338	−2.349669	−0.000100
C	−2.302653	−0.197391	−0.000017
C	−2.322673	−3.073889	−0.000141
C	−3.489413	−0.916709	−0.000060
C	−3.492017	−2.339687	−0.000121
O	5.245661	−1.559375	0.000008
O	3.117224	−2.822958	−0.000072
O	0.301653	1.811044	0.000090
H	3.057264	2.373260	−2.192599
H	3.352921	3.858056	−1.268309
H	1.731658	3.130049	−1.270553
H	3.057112	2.373247	2.192700
H	1.731547	3.130004	1.270570
H	3.352790	3.858056	1.268436
H	0.481067	−3.765652	−0.000138
H	6.420648	0.712550	0.000106
H	5.220682	2.880961	0.000141
H	−2.334734	−4.158458	−0.000187
H	−4.456799	−2.834628	−0.000153
H	−2.305489	0.884687	0.000028
C	−4.829894	−0.242755	−0.000047
O	−4.728138	1.094590	0.000031
C	−5.985994	1.828513	0.000065
H	−6.557276	1.529836	0.883447
H	−6.557276	1.529919	−0.883345

C	−5.652391	3.305773	0.000135
H	−6.578085	3.888477	0.000164
H	−5.076347	3.579595	−0.888153
H	−5.076345	3.579511	0.888447
O	−5.878085	−0.855480	−0.000075
H	4.556379	−2.323061	−0.000040

Table S26. Cartesian coordinates for **CQ3_O1**.

Zero-point correction	0.352282 (Hartree/Particle)
Thermal correction to Energy	0.375646
Thermal correction to Enthalpy	0.376590
Thermal correction to Gibbs Free Energy	0.299104
Sum of electronic and zero-point Energies	−1241.421016
Sum of electronic and thermal Energies	−1241.397651
Sum of electronic and thermal Enthalpies	−1241.396707
Sum of electronic and thermal Free Energies	−1241.474193

Table S27. Cartesian coordinates for **CQ3_O2**.

C	−5.338072	0.774327	−0.000037
C	−4.663842	1.946976	−0.000151
C	−4.597158	−0.443489	0.000002
C	−3.142101	−0.431102	−0.000026
C	−2.460276	0.760788	−0.000084
C	−3.179111	2.085401	−0.000052
C	−2.799176	2.915676	1.280455
C	−2.798774	2.916491	−1.279749
C	−2.454273	−1.764893	−0.000040
C	−0.925136	0.755406	0.000076
C	−1.026280	−1.726370	−0.000079
C	−0.284411	−0.546873	−0.000083
N	−0.178883	−2.800386	−0.000066
C	1.095557	−0.917863	−0.000053
C	1.121382	−2.349663	−0.000095
C	2.302679	−0.197384	−0.000040
C	2.322729	−3.073866	−0.000123
C	3.489441	−0.916686	−0.000071
C	3.492058	−2.339665	−0.000111
O	−5.245758	−1.559327	−0.000035
O	−3.117196	−2.822930	−0.000023
O	−0.301664	1.811000	0.000441
H	−3.057615	2.372503	2.192767
H	−3.353024	3.857641	1.268939
H	−1.731810	3.129524	1.271180
H	−3.056906	2.373944	−2.192528
H	−1.731432	3.130409	−1.270083
H	−3.352665	3.858431	−1.267822
H	−0.481012	−3.765655	−0.000027
H	−6.420630	0.712531	−0.000040
H	−5.220665	2.880932	−0.000280
H	2.334799	−4.158435	−0.000142
H	4.456853	−2.834581	−0.000125
H	2.305510	0.884696	−0.000005

C	4.829895	−0.242706	−0.000060
O	4.728091	1.094592	−0.000041
C	5.985966	1.828571	−0.000039
H	6.557251	1.529861	−0.883397
H	6.557178	1.529972	0.883406
C	5.652257	3.305792	−0.000142
H	6.577914	3.888553	−0.000128
H	5.076155	3.579590	0.888113
H	5.076246	3.579486	−0.888488
O	5.878067	−0.855459	−0.000075
H	−4.556408	−2.322926	0.000004

Table S28. Energies for **CQ3_O2**.

Zero-point correction	0.352283 (Hartree/Particle)
Thermal correction to Energy	0.375649
Thermal correction to Enthalpy	0.376593
Thermal correction to Gibbs Free Energy	0.299099
Sum of electronic and zero-point Energies	−1241.421014
Sum of electronic and thermal Energies	−1241.397649
Sum of electronic and thermal Enthalpies	−1241.396704
Sum of electronic and thermal Free Energies	−1241.474198

Table S29. Cartesian coordinates for **CQ3_O3**.

C	−5.408485	0.726464	−0.039728
C	−4.737357	1.878294	0.074705
C	−4.707751	−0.547647	−0.207625
C	−3.208257	−0.500051	−0.017597
C	−2.521435	0.685404	0.037310
C	−3.238672	2.039566	0.073226
C	−2.881436	2.813951	1.381109
C	−2.891857	2.901556	−1.180582
C	−2.500190	−1.822981	0.087617
C	−1.033466	0.654617	0.049617
C	−1.025605	−1.737664	0.018073
C	−0.305245	−0.534357	0.004102
N	−0.181810	−2.770972	0.043793
C	1.108626	−0.893402	0.006303
C	1.138311	−2.315338	0.032026
C	2.317517	−0.195162	−0.012898
C	2.322429	−3.051259	0.039312
C	3.512177	−0.921954	−0.002592
C	3.509651	−2.330688	0.023789
O	−5.269251	−1.583958	−0.517077
O	−3.030503	−2.901169	0.277790
O	−0.425899	1.825186	0.081395
H	−3.122959	2.218373	2.265684
H	−3.477267	3.729716	1.421502
H	−1.830594	3.098560	1.416547
H	−3.145429	2.369636	−2.101401
H	−1.838339	3.179571	−1.205224
H	−3.480462	3.822587	−1.152806
H	−0.492443	−3.737565	0.074224

H	−6.492292	0.684722	−0.065562
H	−5.285230	2.814544	0.155289
H	2.320175	−4.135818	0.057307
H	4.468156	−2.837252	0.030096
H	2.386948	0.887901	−0.043368
C	4.850815	−0.238670	−0.022213
O	4.735203	1.097759	−0.049951
C	5.990380	1.844999	−0.072209
H	6.557505	1.528699	−0.951609
H	6.565053	1.568750	0.815763
C	5.645912	3.318772	−0.104198
H	6.568270	3.906121	−0.122018
H	5.075188	3.611269	0.781753
H	5.066663	3.570592	−0.997062
O	5.899494	−0.845821	−0.013096
H	0.541002	1.733718	0.076563

Table S30. Energies for **CQ3_O3**.

Zero-point correction	0.351992 (Hartree/Particle)
Thermal correction to Energy	0.375969
Thermal correction to Enthalpy	0.376913
Thermal correction to Gibbs Free Energy	0.297390
Sum of electronic and zero-point Energies	−1241.380729
Sum of electronic and thermal Energies	−1241.356752
Sum of electronic and thermal Enthalpies	−1241.355808
Sum of electronic and thermal Free Energies	−1241.435331

Table S31. Cartesian coordinates for **CQ3_N4**.

C	−5.380552	0.793330	0.019729
C	−4.695556	1.936410	0.122430
C	−4.696243	−0.482960	−0.184363
C	−3.194570	−0.452405	−0.002971
C	−2.501521	0.722813	0.032928
C	−3.198876	2.075662	0.075503
C	−2.772429	2.855627	1.357785
C	−2.858441	2.918349	−1.192345
C	−2.507833	−1.779768	0.112848
C	−0.988286	0.726601	0.004126
C	−1.056525	−1.710182	−0.048324
C	−0.305921	−0.603017	−0.044810
N	−0.194584	−2.915331	0.004048
C	1.109856	−0.958076	−0.024786
C	1.191351	−2.357923	0.006244
C	2.283529	−0.208942	−0.028280
C	2.371158	−3.065445	0.023877
C	3.500927	−0.899704	−0.006030
C	3.541997	−2.299342	0.019140
O	−5.262663	−1.500483	−0.526487
O	−3.025280	−2.844147	0.388368
O	−0.312949	1.733630	−0.000333
H	−2.992485	2.278521	2.258888
H	−3.340464	3.786792	1.409685

H	−1.713270	3.103970	1.337052
H	−3.141542	2.386691	−2.103480
H	−1.797724	3.160656	−1.228829
H	−3.421396	3.853660	−1.161238
H	−0.414542	−3.470740	0.842100
H	−6.463825	0.763672	0.019852
H	−5.232144	2.875841	0.223394
H	2.411460	−4.147964	0.045431
H	4.511340	−2.782256	0.036908
H	2.257597	0.871229	−0.049237
C	4.826083	−0.176677	−0.008256
O	4.671411	1.146160	−0.035129
C	5.896464	1.952012	−0.039444
H	6.482842	1.660989	−0.912391
H	6.465310	1.696902	0.856060
C	5.488159	3.407160	−0.073037
H	6.385350	4.030853	−0.076659
H	4.894464	3.672642	0.804159
H	4.912383	3.636633	−0.972060
O	5.881353	−0.760424	0.012490
H	−0.372872	−3.529808	−0.801019

Table S32. Energies for **CQ3_N4**.

Zero-point correction	0.352328 (Hartree/Particle)
Thermal correction to Energy	0.376335
Thermal correction to Enthalpy	0.377279
Thermal correction to Gibbs Free Energy	0.297641
Sum of electronic and zero-point Energies	−1241.346918
Sum of electronic and thermal Energies	−1241.322911
Sum of electronic and thermal Enthalpies	−1241.321966
Sum of electronic and thermal Free Energies	−1241.401605

Table S33. Cartesian coordinates for **CQ3_O5**.

C	−5.416776	0.718174	−0.011017
C	−4.758574	1.872553	0.120572
C	−4.699699	−0.538898	−0.238292
C	−3.206918	−0.490127	−0.017281
C	−2.538185	0.697692	0.043681
C	−3.263943	2.037001	0.088791
C	−2.866094	2.815343	1.379922
C	−2.928972	2.896191	−1.168308
C	−2.502198	−1.811524	0.116503
C	−1.016627	0.731619	0.041098
C	−1.034259	−1.730411	0.031067
C	−0.331435	−0.560017	0.008746
N	−0.175495	−2.803971	0.055994
C	1.064092	−0.913624	0.008291
C	1.110924	−2.347331	0.035517
C	2.243164	−0.189292	−0.010088
C	2.322261	−3.056591	0.036778
C	3.461924	−0.900221	−0.006266
C	3.488254	−2.327784	0.016841

O	−5.249728	−1.547573	−0.633252
O	−3.036966	−2.875497	0.345182
O	−0.378230	1.772725	0.042324
H	−3.084983	2.225658	2.273327
H	−3.451545	3.736072	1.434427
H	−1.810527	3.078146	1.371183
H	−3.197070	2.365744	−2.084801
H	−1.870916	3.150145	−1.194074
H	−3.506883	3.822885	−1.135361
H	−0.486665	−3.766755	0.092992
H	−6.499104	0.663932	−0.025935
H	−5.314046	2.800053	0.232505
H	2.343478	−4.139107	0.054624
H	4.439999	−2.841345	0.018844
H	2.219793	0.891703	−0.026768
C	4.689150	−0.174407	−0.027232
O	4.670467	1.119754	−0.050283
C	5.902476	1.939341	−0.074194
H	6.462842	1.666862	−0.973275
H	6.474331	1.702018	0.827589
C	5.478084	3.388582	−0.100023
H	6.373408	4.014334	−0.119038
H	4.900033	3.642866	0.789160
H	4.887308	3.607235	−0.990328
O	5.814426	−0.848698	−0.022703
H	6.609368	−0.295953	−0.037800

Table S34. Energies for **CQ3_O5**.

Zero-point correction	0.352173 (Hartree/Particle)
Thermal correction to Energy	0.376133
Thermal correction to Enthalpy	0.377078
Thermal correction to Gibbs Free Energy	0.298142
Sum of electronic and zero-point Energies	−1241.380763
Sum of electronic and thermal Energies	−1241.356802
Sum of electronic and thermal Enthalpies	−1241.355858
Sum of electronic and thermal Free Energies	−1241.434793

Table S35. Cartesian coordinates for **CQ3_O6**.

C	−5.321300	0.880819	−0.031144
C	−4.606354	2.003338	0.079603
C	−4.668062	−0.415235	−0.227498
C	−3.173960	−0.435478	−0.006248
C	−2.446922	0.718731	0.032908
C	−3.105203	2.092893	0.052270
C	−2.675229	2.872155	1.332737
C	−2.722168	2.912762	−1.217393
C	−2.536638	−1.787690	0.150203
C	−0.926820	0.676695	0.032038
C	−1.065806	−1.782682	0.052114
C	−0.306883	−0.649187	0.007429
N	−0.261125	−2.897374	0.088818
C	1.068686	−1.071171	0.005477

C	1.044816	−2.506013	0.053562
C	2.278172	−0.399605	−0.035063
C	2.218572	−3.275772	0.051448
C	3.454515	−1.175179	−0.029300
C	3.420043	−2.610166	0.007344
O	−5.265765	−1.405220	−0.598926
O	−3.120388	−2.818965	0.404607
O	−0.233842	1.682024	0.030113
H	−2.930541	2.310989	2.234780
H	−3.212195	3.822856	1.366607
H	−1.607464	3.080026	1.327174
H	−3.010691	2.380211	−2.126401
H	−1.653082	3.115957	−1.241606
H	−3.255022	3.866389	−1.203707
H	−0.619745	−3.843217	0.140721
H	−6.404912	0.880783	−0.049211
H	−5.115395	2.959302	0.170517
H	2.188796	−4.357891	0.083103
H	4.349105	−3.165647	0.004097
H	2.313658	0.677376	−0.090071
C	4.753011	−0.640904	−0.097007
O	4.484952	1.345908	0.201291
C	5.568612	2.172666	−0.351168
H	5.448488	2.091894	−1.430930
H	6.523880	1.726979	−0.063991
C	5.441421	3.604237	0.127785
H	6.241741	4.200550	−0.317242
H	5.548660	3.676812	1.214553
H	4.485204	4.039566	−0.167205
O	5.882408	−0.781458	−0.232400
H	4.403489	1.529089	1.149045

Table S36. Energies for **CQ3_O6**.

Zero-point correction	0.350340 (Hartree/Particle)
Thermal correction to Energy	0.375049
Thermal correction to Enthalpy	0.375993
Thermal correction to Gibbs Free Energy	0.294573
Sum of electronic and zero-point Energies	−1241.352664
Sum of electronic and thermal Energies	−1241.327955
Sum of electronic and thermal Enthalpies	−1241.327011
Sum of electronic and thermal Free Energies	−1241.408431

1.5. Protonated Species for CQ4

Table S37. Cartesian coordinates for **CQ4_O1**.

C	5.248891	−0.251905	−0.000248
C	4.753188	−1.510387	−0.000053
C	4.338026	0.844427	−0.000380
C	2.900557	0.620605	−0.000295
C	2.399354	−0.659222	−0.000097
C	3.304718	−1.864259	0.000018
C	3.048857	−2.741636	1.279821

C	3.048829	−2.741862	−1.279640
C	2.028174	1.840309	−0.000439
C	0.881665	−0.878595	0.000082
C	0.620718	1.592762	−0.000267
C	0.060943	0.316278	−0.000030
N	−0.374565	2.530093	−0.000296
C	−1.358732	0.481157	0.000027
C	−1.593884	1.892646	−0.000135
C	−2.442537	−0.412840	0.000212
C	−2.890378	2.428667	−0.000116
C	−3.715228	0.131048	0.000230
C	−3.942701	1.532890	0.000069
O	4.817969	2.042866	−0.000514
O	2.528157	2.984545	−0.000583
O	0.415129	−2.013580	0.000183
H	3.220226	−2.165921	2.192694
H	3.736418	−3.591004	1.271052
H	2.025004	−3.111318	1.266498
H	3.220147	−2.166292	−2.192614
H	2.024986	−3.111568	−1.266206
H	3.736414	−3.591209	−1.270755
H	−0.216725	3.529238	−0.000439
H	6.310788	−0.032590	−0.000299
H	5.440619	−2.352949	0.000053
H	−3.067049	3.498787	−0.000237
H	−4.963576	1.896533	0.000097
H	−2.280589	−1.482954	0.000342
Br	−5.223541	−1.015987	0.000490
H	4.026426	2.698995	−0.000522

Table S38. Energies for **CQ4_O1**.

Zero-point correction	0.271054 (Hartree/Particle)
Thermal correction to Energy	0.289893
Thermal correction to Enthalpy	0.290837
Thermal correction to Gibbs Free Energy	0.223764
Sum of electronic and zero-point Energies	−3545.399576
Sum of electronic and thermal Energies	−3545.380738
Sum of electronic and thermal Enthalpies	−3545.379794
Sum of electronic and thermal Free Energies	−3545.446867

Table S39. Cartesian coordinates for **CQ4_O2**.

C	−5.255668	−0.229803	−0.000331
C	−4.770181	−1.482842	−0.000075
C	−4.349101	0.888947	−0.000099
C	−2.893559	0.608147	−0.000028
C	−2.404623	−0.667610	−0.000091
C	−3.321967	−1.864999	0.000052
C	−3.077680	−2.738482	−1.277690
C	−3.077505	−2.737626	1.278474
C	−2.010219	1.791021	0.000054
C	−0.888878	−0.890706	−0.000185
C	−0.621635	1.586235	−0.000025

C	−0.056947	0.309524	−0.000070
N	0.378276	2.529562	−0.000142
C	1.352483	0.474384	0.000017
C	1.588300	1.890959	0.000024
C	2.438905	−0.420474	0.000020
C	2.886119	2.423884	0.000051
C	3.706055	0.121557	0.000035
C	3.932357	1.528762	0.000059
O	−4.747566	2.072723	0.000023
O	−2.516478	2.970222	0.000058
O	−0.415379	−2.012253	−0.000339
H	−3.229875	−2.155519	−2.188317
H	−3.788035	−3.567452	−1.280385
H	−2.068338	−3.143370	−1.269581
H	−3.229600	−2.153997	2.188673
H	−2.068154	−3.142482	1.270374
H	−3.787847	−3.566603	1.281754
H	0.231486	3.530038	0.000084
H	−6.316535	−0.011165	−0.000553
H	−5.462529	−2.319833	0.000069
H	3.065486	3.491868	0.000076
H	4.951606	1.893054	0.000058
H	2.273915	−1.488580	−0.000013
Br	5.221283	−1.019790	−0.000012
H	−3.586555	2.828940	0.000077

Table S40. Energies for **CQ4_O2**.

Zero-point correction	0.271053 (Hartree/Particle)
Thermal correction to Energy	0.289892
Thermal correction to Enthalpy	0.290837
Thermal correction to Gibbs Free Energy	0.223757
Sum of electronic and zero-point Energies	−3545.399578
Sum of electronic and thermal Energies	−3545.380738
Sum of electronic and thermal Enthalpies	−3545.379794
Sum of electronic and thermal Free Energies	−3545.446874

Table S41. Cartesian coordinates for **CQ4_O3**.

C	−5.309155	−0.181602	−0.020284
C	−4.818090	−1.416637	0.112050
C	−4.420932	0.956240	−0.249974
C	−2.952206	0.690851	−0.015389
C	−2.449325	−0.579097	0.036275
C	−3.363493	−1.806878	0.078415
C	−3.179675	−2.702999	−1.185397
C	−3.102926	−2.635841	1.374355
C	−2.066515	1.894956	0.137691
C	−0.974817	−0.764420	0.035215
C	−0.620864	1.603547	0.048670
C	−0.085175	0.307467	0.005022
N	0.364218	2.499373	0.090617
C	1.365806	0.457900	0.003906
C	1.601370	1.856787	0.059875

C	2.458560	−0.409003	−0.038490
C	2.879825	2.406681	0.074428
C	3.740101	0.141847	−0.019986
C	3.953933	1.529241	0.035959
O	−4.798812	2.035962	−0.652750
O	−2.447085	3.014193	0.394557
O	−0.547444	−2.010642	0.035426
H	−3.349388	−2.128384	−2.098378
H	−3.912834	−3.511573	−1.158017
H	−2.188965	−3.152017	−1.222955
H	−3.227932	−2.017398	2.265973
H	−2.110803	−3.081255	1.383999
H	−3.832441	−3.446599	1.426133
H	0.204662	3.500928	0.142248
H	−6.373362	0.022102	−0.032854
H	−5.500090	−2.254695	0.224416
H	3.040251	3.477178	0.115540
H	4.965375	1.913101	0.047590
H	2.362172	−1.487074	−0.092577
Br	5.238954	−1.019138	−0.077351
H	0.418642	−2.070435	0.019940

Table S42. Energies for **CQ4_O3**.

Zero-point correction	0.270820 (Hartree/Particle)
Thermal correction to Energy	0.290267
Thermal correction to Enthalpy	0.291211
Thermal correction to Gibbs Free Energy	0.222017
Sum of electronic and zero-point Energies	−3545.358193
Sum of electronic and thermal Energies	−3545.338746
Sum of electronic and thermal Enthalpies	−3545.337802
Sum of electronic and thermal Free Energies	−3545.406997

Table S43. Cartesian coordinates for **CQ4_N4**.

C	−5.304089	−0.270134	0.042450
C	−4.791258	−1.500962	0.135924
C	−4.443398	0.892753	−0.169998
C	−2.960797	0.643900	0.001089
C	−2.445119	−0.619746	0.025407
C	−3.331650	−1.856879	0.066155
C	−3.134071	−2.724293	−1.215654
C	−3.004903	−2.705683	1.333365
C	−2.090366	1.857484	0.123027
C	−0.948821	−0.841736	−0.016298
C	−0.663351	1.580944	−0.042368
C	−0.082672	0.376647	−0.050940
N	0.364154	2.648651	0.019159
C	1.370105	0.523706	−0.030280
C	1.652679	1.896138	0.014199
C	2.415623	−0.393933	−0.042386
C	2.927128	2.415442	0.037119
C	3.718644	0.113637	−0.014669
C	3.976345	1.489198	0.023742

O	−4.856902	1.981866	−0.510483
O	−2.447452	2.983178	0.408213
O	−0.423717	−1.934501	−0.042904
H	−3.344753	−2.144223	−2.116804
H	−3.829075	−3.566180	−1.188262
H	−2.121321	−3.120426	−1.267933
H	−3.127691	−2.114949	2.244115
H	−1.992776	−3.102945	1.293731
H	−3.699219	−3.547092	1.384426
H	0.224134	3.222060	0.862455
H	−6.371450	−0.083329	0.056140
H	−5.457663	−2.352475	0.243078
H	3.132354	3.478872	0.069110
H	4.998577	1.843413	0.045281
H	2.220604	−1.456689	−0.074299
Br	5.177420	−1.096840	−0.030366
H	0.273887	3.289556	−0.780252

Table S44. Energies for **CQ4_N4**.

Zero-point correction	0.271077 (Hartree/Particle)
Thermal correction to Energy	0.290559
Thermal correction to Enthalpy	0.291503
Thermal correction to Gibbs Free Energy	0.222218
Sum of electronic and zero-point Energies	−3545.323935
Sum of electronic and thermal Energies	−3545.304453
Sum of electronic and thermal Enthalpies	−3545.303509
Sum of electronic and thermal Free Energies	−3545.372794

1.6. Fragments for CQ1

m/*z* 292a corresponds to **CQ1_O1** and *m*/*z* 292c corresponds to **CQ1_O2**.

Table S45. Cartesian coordinates for *m*/*z* 292b.

C	−2.748930	1.492265	−0.145775
C	−1.464831	0.847832	−0.105418
C	−1.320789	−0.576581	−0.150941
C	−2.424830	−2.877511	−0.405452
C	−0.266822	1.756528	−0.033743
C	0.071001	−1.197255	−0.111259
C	1.004970	1.108022	−0.003428
C	1.193684	−0.275636	−0.039285
N	2.215338	1.734014	0.064486
C	2.595900	−0.510263	0.012317
C	3.214395	0.781543	0.076577
C	3.399013	−1.670867	0.010446
C	4.602654	0.938080	0.138336
C	4.769425	−1.510782	0.072032
C	5.361867	−0.222394	0.135005
O	−2.910115	2.811280	−0.130631
O	−0.391000	2.995123	−0.005980
O	0.223951	−2.412471	−0.128834
H	−1.777792	−3.166127	−1.237732
H	−3.428603	−3.270024	−0.577499

H	−1.986357	−3.366797	0.470565
H	2.332191	2.737762	0.100326
H	6.443239	−0.144705	0.181727
H	2.937499	−2.649622	−0.037686
H	5.413922	−2.383254	0.072683
H	5.065180	1.917917	0.186536
H	−2.010854	3.231937	−0.083669
C	−4.584353	−1.285714	1.104175
H	−4.083203	−0.945114	2.011332
H	−5.600932	−0.890653	1.083744
H	−4.626684	−2.374650	1.098027
C	−2.450552	−1.390084	−0.242698
C	−3.873308	0.693816	−0.194708
H	−4.847791	1.173733	−0.198435
C	−3.800240	−0.768171	−0.177913
H	−4.389396	−1.156917	−1.027377

Table S46. Energies for *m*/*z* 292b.

Zero-point correction	0.280268 (Hartree/Particle)
Thermal correction to Energy	0.297885
Thermal correction to Enthalpy	0.298830
Thermal correction to Gibbs Free Energy	0.235073
Sum of electronic and zero-point Energies	−974.270142
Sum of electronic and thermal Energies	−974.252525
Sum of electronic and thermal Enthalpies	−974.251581
Sum of electronic and thermal Free Energies	−974.315337

Table S47. Cartesian coordinates for *m*/*z* 292d.

C	3.448074	0.666495	1.382189
C	3.702417	−0.470553	0.720204
C	2.246723	1.449200	1.105196
C	1.590348	1.011106	−0.419233
C	1.519443	−0.414937	−0.328252
C	2.841700	−1.140722	−0.364847
C	2.830971	−2.660811	−0.072123
C	3.548231	−0.926659	−1.747434
C	0.328254	1.835900	−0.679293
C	0.197845	−1.090002	−0.004793
C	−0.904426	1.120561	−0.420938
C	−0.970357	−0.244663	−0.106851
N	−2.153903	1.638086	−0.459297
C	−2.357056	−0.566810	0.057989
C	−3.076964	0.641797	−0.168337
C	−3.055802	−1.743991	0.373033
C	−4.469035	0.709286	−0.090697
C	−4.442355	−1.678359	0.450494
C	−5.136138	−0.471935	0.222183
O	1.726041	2.331974	1.702351
O	0.410996	2.980934	−1.078941
O	0.153784	−2.281697	0.294892
H	2.433708	−2.895555	0.914037
H	3.861275	−3.022272	−0.136857

H	2.231753	−3.196343	−0.808671
H	3.681792	0.124301	−2.008530
H	2.972524	−1.419715	−2.534965
H	4.534541	−1.395062	−1.707238
H	−2.356292	2.606854	−0.673748
H	4.096783	1.047884	2.164506
H	4.591324	−1.042237	0.978716
H	−6.218674	−0.462990	0.293222
H	−2.518089	−2.667570	0.550571
H	−5.006898	−2.572454	0.692436
H	−5.008266	1.634325	−0.264437
H	2.404033	1.395129	−1.041302

Table S48. Energies for *m*/*z* 292d.

Zero-point correction	0.278804 (Hartree/Particle)
Thermal correction to Energy	0.296873
Thermal correction to Enthalpy	0.297817
Thermal correction to Gibbs Free Energy	0.232873
Sum of electronic and zero-point Energies	−974.211489
Sum of electronic and thermal Energies	−974.193420
Sum of electronic and thermal Enthalpies	−974.192476
Sum of electronic and thermal Free Energies	−974.257420

Table S49. Cartesian coordinates for *m*/*z* 292e.

C	−3.978308	0.902536	−0.293301
C	−3.950330	−0.434059	−0.193232
C	−2.756819	1.703083	−0.203538
C	−1.474546	0.908528	−0.069623
C	−1.457528	−0.447916	0.081893
C	−2.723773	−1.286669	−0.004533
C	−2.642947	−2.252071	−1.230277
C	−2.916493	−2.114741	1.301793
C	−0.217695	1.709030	−0.116720
C	−0.143821	−1.159396	0.302716
C	0.885033	1.130212	0.891639
C	1.036703	−0.285221	0.504047
N	2.201801	1.673133	0.765652
C	2.350613	−0.515406	0.145068
C	3.061160	0.746171	0.309674
C	3.038758	−1.665034	−0.352193
C	4.429895	0.858528	−0.004353
C	4.369095	−1.533459	−0.640511
C	5.051226	−0.280624	−0.467300
O	−2.735439	2.921949	−0.200289
O	0.009560	2.681237	−0.774603
O	−0.015818	−2.373896	0.294662
H	−2.502410	−1.694404	−2.160115
H	−3.582986	−2.805432	−1.306852
H	−1.830221	−2.967589	−1.109077
H	−2.985816	−1.462433	2.176792
H	−2.098471	−2.822215	1.440592
H	−3.848974	−2.681278	1.229885

H	2.419431	2.650849	0.910691
H	−4.902355	1.455714	−0.424963
H	−4.881889	−0.993439	−0.249541
H	6.106508	−0.234340	−0.718581
H	2.505740	−2.600735	−0.468311
H	4.934216	−2.382809	−1.007962
H	4.964785	1.794300	0.111201
H	0.462063	1.296603	1.891039

Table S50. Energies for *m*/*z* 292e.

Zero-point correction	0.279897 (Hartree/Particle)
Thermal correction to Energy	0.297823
Thermal correction to Enthalpy	0.298767
Thermal correction to Gibbs Free Energy	0.234465
Sum of electronic and zero-point Energies	−974.228196
Sum of electronic and thermal Energies	−974.210270
Sum of electronic and thermal Enthalpies	−974.209326
Sum of electronic and thermal Free Energies	−974.273628

Table S51. Cartesian coordinates for *m*/*z* 292f.

C	3.139187	−2.119839	−0.000207
C	4.259204	−1.379823	−0.000015
C	1.801648	−1.525958	−0.000183
C	1.723198	−0.037885	−0.000076
C	2.879962	0.679743	0.000140
C	4.259582	0.118028	0.000174
C	5.003610	0.640735	−1.268477
C	5.003594	0.640505	1.268875
C	0.470835	0.781515	−0.000156
C	−1.884540	2.516081	−0.000080
C	−0.922092	0.265183	−0.000089
C	−2.026273	1.161622	−0.000043
N	−1.390124	−0.981135	−0.000039
C	−3.237021	0.355806	0.000063
C	−2.778329	−0.989398	−0.000004
C	−4.608274	0.627139	0.000170
C	−3.656026	−2.078036	0.000013
C	−5.480606	−0.455665	0.000191
C	−5.012839	−1.787245	0.000114
O	0.813024	−2.277798	−0.000168
O	0.523058	2.014714	−0.000235
O	−1.960337	3.660836	−0.000088
H	4.514178	0.303268	−2.185142
H	6.031339	0.267304	−1.266186
H	5.037585	1.733649	−1.268131
H	4.514169	0.302894	2.185494
H	5.037576	1.733421	1.268734
H	6.031327	0.267086	1.266551
H	−0.685234	−1.762403	−0.000111
H	3.161104	−3.204516	−0.000332
H	5.231986	−1.866772	0.000006
H	−5.731248	−2.599764	0.000142

H	−4.985294	1.644309	0.000208
H	−6.550013	−0.273486	0.000265
H	−3.290131	−3.098882	−0.000033
H	2.798656	1.764764	0.000267

Table S52. Energies for *m*/*z* 292f.

Zero-point correction	0.278849 (Hartree/Particle)
Thermal correction to Energy	0.297334
Thermal correction to Enthalpy	0.298278
Thermal correction to Gibbs Free Energy	0.231759
Sum of electronic and zero-point Energies	−974.266410
Sum of electronic and thermal Energies	−974.247926
Sum of electronic and thermal Enthalpies	−974.246981
Sum of electronic and thermal Free Energies	−974.313500

Table S53. Cartesian coordinates for *m*/*z* 292g.

C	−3.231412	−1.455787	−0.721208
C	−3.113321	−0.303853	−1.374417
C	−2.448801	−1.724366	0.472652
C	−1.456218	−0.668155	0.827306
C	−1.298919	0.503587	0.153152
C	−2.240699	0.862228	−0.974024
C	−3.195689	2.002090	−0.467497
C	−1.440341	1.378556	−2.198513
C	−0.647255	−1.006433	1.919495
C	−0.226914	1.489389	0.578251
C	2.142397	1.935296	0.976230
C	1.152098	1.139614	0.426572
N	3.343996	1.414680	0.706918
C	1.836225	0.040563	−0.259004
C	3.205573	0.252855	−0.050638
C	1.448157	−1.063176	−1.029217
C	4.190142	−0.585908	−0.554552
C	2.419015	−1.903654	−1.535395
C	3.779610	−1.671925	−1.298263
O	−2.512716	−2.726478	1.162528
O	−0.036613	−1.309514	2.805945
O	−0.637009	2.525088	1.095989
H	−3.763093	1.682188	0.395390
H	−3.886479	2.231029	−1.267989
H	−2.641082	2.890566	−0.215225
H	−0.759341	0.631726	−2.583772
H	−0.880899	2.269664	−1.948483
H	−2.135681	1.637269	−2.985545
H	4.209351	1.803552	0.999185
H	−3.900249	−2.230568	−1.035101
H	−3.708312	−0.132019	−2.251431
H	4.508284	−2.343505	−1.705494
H	0.419787	−1.268403	−1.250220
H	2.128129	−2.749167	−2.126693
H	5.230224	−0.395778	−0.376422
H	2.020203	2.832385	1.542100

Table S54. Energies for *m*/*z* 292g.

Zero-point correction	0.278021 (Hartree/Particle)
Thermal correction to Energy	0.297156
Thermal correction to Enthalpy	0.298101
Thermal correction to Gibbs Free Energy	0.229406
Sum of electronic and zero-point Energies	−974.219194
Sum of electronic and thermal Energies	−974.200059
Sum of electronic and thermal Enthalpies	−974.199115
Sum of electronic and thermal Free Energies	−974.267810

Table S55. Cartesian coordinates for TS *m*/*z* 292g.

C	−4.828713	0.154314	0.100241
C	−4.115619	1.285772	−0.067979
C	−4.209707	−1.183211	0.142292
C	−2.724634	−1.054727	0.021050
C	−1.935086	0.047209	−0.138183
C	−2.609138	1.405875	−0.248067
C	−2.043952	2.383385	0.812192
C	−2.322278	1.969751	−1.668377
C	−1.536476	−1.715519	−0.107510
C	0.707650	0.153890	1.252750
C	1.305256	−0.099088	−1.144385
C	1.596291	0.063461	0.227245
N	2.457478	−0.194119	−1.803294
C	3.058966	0.054061	0.373705
C	3.558373	−0.109127	−0.935450
C	3.941677	0.165186	1.448897
C	4.921307	−0.165469	−1.218451
C	5.306251	0.108797	1.175118
C	5.788768	−0.053516	−0.135921
O	−4.794018	−2.241829	0.257834
O	−0.613964	−2.433097	−0.207031
O	0.167014	0.247589	2.262624
H	−2.188377	2.005963	1.827603
H	−2.553066	3.348455	0.733644
H	−0.976254	2.554680	0.645691
H	−2.695505	1.300982	−2.448242
H	−1.247351	2.117826	−1.809768
H	−2.815968	2.938805	−1.787490
H	2.525573	−0.324478	−2.805504
H	−5.908530	0.182091	0.209808
H	−4.651352	2.234596	−0.092335
H	6.858826	−0.092883	−0.307655
H	3.585183	0.289610	2.466087
H	6.013771	0.191620	1.993046
H	5.290429	−0.291877	−2.230679
H	0.334412	−0.144115	−1.613571

Table S56. Energies for TS *m*/*z* 292g.

Zero-point correction	0.274406 (Hartree/Particle)
Thermal correction to Energy	0.294354
Thermal correction to Enthalpy	0.295298
Thermal correction to Gibbs Free Energy	0.221997
Sum of electronic and zero-point Energies	−974.137502
Sum of electronic and thermal Energies	−974.117555
Sum of electronic and thermal Enthalpies	−974.116611
Sum of electronic and thermal Free Energies	−974.189912

Table S57. Cartesian coordinates for TS *m*/*z* 292f.

C	5.228810	0.951887	−0.222219
C	5.348728	−0.376575	−0.065849
C	3.930250	1.629462	−0.217583
C	2.744765	0.676133	−0.021061
C	2.878166	−0.671421	0.137332
C	4.205903	−1.338575	0.129841
C	4.372802	−2.087069	1.491517
C	4.199459	−2.389653	−1.027604
C	1.514970	1.341789	−0.002474
C	−1.224876	−0.512383	−0.081639
C	−2.242299	0.999020	0.258714
C	−2.543704	−0.363997	−0.025431
N	−3.454826	1.577290	0.368226
C	−3.966088	−0.621205	−0.091167
C	−4.515784	0.658288	0.172358
C	−4.799674	−1.715840	−0.324657
C	−5.890968	0.868090	0.209324
C	−6.177205	−1.506961	−0.289093
C	−6.711680	−0.235848	−0.026098
O	3.743899	2.821211	−0.351184
O	0.657677	2.085220	0.009452
O	−0.064567	−0.759749	−0.152840
H	4.376736	−1.390502	2.332967
H	5.324075	−2.625940	1.483290
H	3.569362	−2.814896	1.633925
H	4.074554	−1.911105	−2.001512
H	3.398386	−3.119605	−0.883112
H	5.153504	−2.923779	−1.023130
H	−3.601431	2.557648	0.569089
H	6.088921	1.598966	−0.360875
H	6.338653	−0.827072	−0.077731
H	−7.788916	−0.106811	−0.005309
H	−4.389513	−2.699267	−0.527298
H	−6.848632	−2.340219	−0.467184
H	−6.312865	1.847216	0.411257
H	1.995425	−1.289965	0.269353

Table S58. Energies for TS *m*/*z* 292f.

Zero-point correction	0.273587 (Hartree/Particle)
Thermal correction to Energy	0.293830
Thermal correction to Enthalpy	0.294774
Thermal correction to Gibbs Free Energy	0.219791
Sum of electronic and zero-point Energies	−974.120827
Sum of electronic and thermal Energies	−974.100585
Sum of electronic and thermal Enthalpies	−974.099640
Sum of electronic and thermal Free Energies	−974.174624

Table S59. Cartesian coordinates for product with *m*/*z* 149.

C	−4.913100	−1.132324	−0.000498
C	−5.236650	0.168157	−0.000283
C	−3.518262	−1.606363	−0.000388
C	−2.472180	−0.513865	0.000047
C	−2.823361	0.794109	0.000286
C	−4.234678	1.290754	0.000091
C	−4.441170	2.172182	1.267614
C	−4.440622	2.172526	−1.267296
C	−1.078129	−0.946273	0.000366
C	1.109355	0.061632	0.000167
C	2.141918	−0.842487	0.000191
C	2.386115	0.556216	−0.000056
N	3.276634	−1.536736	0.000205
C	3.805815	0.731035	−0.000096
C	4.343557	−0.597558	0.000054
C	4.665181	1.840776	−0.000271
C	5.713544	−0.833604	0.000012
C	6.031898	1.601324	−0.000288
C	6.541916	0.287971	−0.000153
O	−3.224256	−2.789115	−0.000671
O	−0.557504	−2.007326	0.000797
O	−0.158817	0.297175	0.000038
H	−4.302412	1.591059	2.182643
H	−5.456848	2.577845	1.268600
H	−3.741034	3.013185	1.274101
H	−4.301498	1.591635	−2.182415
H	−3.740457	3.013507	−1.273254
H	−5.456289	2.578215	−1.268585
H	3.369892	−2.544975	0.000513
H	−5.665329	−1.914485	−0.000775
H	−6.282752	0.467854	−0.000382
H	7.617246	0.142189	−0.000180
H	4.266775	2.848930	−0.000390
H	6.723200	2.436728	−0.000413
H	6.124819	−1.836993	0.000109
H	−2.054024	1.561336	0.000668

Table S60. Energies for product with *m*/*z* 149.

Zero-point correction	0.277506 (Hartree/Particle)
Thermal correction to Energy	0.296520
Thermal correction to Enthalpy	0.297464
Thermal correction to Gibbs Free Energy	0.227290
Sum of electronic and zero-point Energies	−974.149160
Sum of electronic and thermal Energies	−974.130146
Sum of electronic and thermal Enthalpies	−974.129202
Sum of electronic and thermal Free Energies	−974.199376

Table S61. Cartesian coordinates for product with *m*/*z* 144.

C	4.636945	1.585277	−0.009146
C	5.100547	0.314430	−0.005353
C	3.206580	1.981144	−0.007623
C	2.365033	0.727483	−0.001517
C	2.866590	−0.534265	0.002207
C	4.287023	−0.978558	0.001378
C	4.600083	−1.805844	1.277460
C	4.595513	−1.816388	−1.268885
C	1.483708	−0.348842	0.004125
C	−0.910125	0.290778	0.008271
C	−2.278342	−1.825129	−0.003814
C	−2.111671	−0.436517	0.002746
N	−3.595527	−2.098927	−0.008223
C	−3.438637	0.160272	0.002058
C	−4.348370	−0.919860	−0.004859
C	−3.923665	1.473399	0.006575
C	−5.731799	−0.741204	−0.007448
C	−5.301689	1.660906	0.004057
C	−6.193032	0.570750	−0.002844
O	2.774854	3.109647	−0.010764
O	0.319223	−0.833631	0.007753
O	−0.561427	1.418724	0.013101
H	4.380160	−1.242720	2.187502
H	5.662057	−2.065111	1.282604
H	4.019612	−2.732005	1.281759
H	4.371828	−1.261045	−2.182795
H	4.015480	−2.742820	−1.263155
H	5.657566	−2.075273	−1.275990
H	−3.984795	−3.031512	−0.013336
H	5.334430	2.417555	−0.013675
H	6.178907	0.164126	−0.006974
H	−7.261645	0.757443	−0.004619
H	−3.242764	2.317437	0.011910
H	−5.701197	2.669589	0.007493
H	−6.417084	−1.582500	−0.012752
H	−1.533226	−2.605592	−0.005454

Table S62. Energies for product with *m*/*z* 144.

Zero-point correction	0.277715 (Hartree/Particle)
Thermal correction to Energy	0.296995
Thermal correction to Enthalpy	0.297939
Thermal correction to Gibbs Free Energy	0.227777
Sum of electronic and zero-point Energies	−974.179513
Sum of electronic and thermal Energies	−974.160233
Sum of electronic and thermal Enthalpies	−974.159289
Sum of electronic and thermal Free Energies	−974.229451

Table S63. Cartesian coordinates for *m*/*z* 274.

C	2.722145	1.612028	0.000023
C	1.465036	1.236228	−0.000109
C	1.563602	−0.219149	−0.000090
C	2.864143	−2.419612	−0.000054
C	0.193534	2.071879	−0.000119
C	0.190662	−0.949583	−0.000116
C	−0.991594	1.259198	−0.000081
C	−1.005140	−0.135529	−0.000068
N	−2.268616	1.727917	−0.000050
C	−2.377870	−0.540713	0.000003
C	−3.145755	0.661304	0.000004
C	−3.036225	−1.783138	0.000057
C	−4.543886	0.654379	0.000060
C	−4.418028	−1.786799	0.000112
C	−5.161608	−0.583227	0.000114
O	0.247693	3.281832	−0.000069
O	0.180058	−2.161124	0.000068
H	2.359217	−2.835122	0.872892
H	3.893872	−2.764589	−0.000091
H	2.359157	−2.835100	−0.872972
H	−2.514423	2.708884	−0.000061
H	−6.243767	−0.634115	0.000158
H	−2.468663	−2.704169	0.000052
H	−4.950274	−2.730158	0.000154
H	−5.117463	1.573187	0.000061
C	5.382651	−0.835906	0.000124
H	5.508746	−1.466106	−0.884086
H	6.175780	−0.089754	0.000192
H	5.508639	−1.466148	0.884320
C	2.793895	−0.910650	−0.000024
C	3.994744	1.241019	0.000053
H	4.877856	1.864552	0.000022
C	4.021300	−0.185471	0.000065

Table S64. Energies for *m*/*z* 274.

Zero-point correction	0.250142 (Hartree/Particle)
Thermal correction to Energy	0.267113
Thermal correction to Enthalpy	0.268057
Thermal correction to Gibbs Free Energy	0.205828
Sum of electronic and zero-point Energies	−897.782219
Sum of electronic and thermal Energies	−897.765248
Sum of electronic and thermal Enthalpies	−897.764304
Sum of electronic and thermal Free Energies	−897.826532

Table S65. Cartesian coordinates for *m*/*z* 264a.

C	−3.686246	0.736895	−0.000110
C	−2.877512	1.816743	−0.000052
C	−3.102719	−0.584324	−0.000103
C	−1.719387	−0.715445	−0.000037
C	−0.840370	0.378942	0.000030
C	−1.365338	1.782878	0.000028
C	−0.862188	2.526806	1.277074
C	−0.862048	2.526848	−1.276939
C	−0.956251	−1.992561	−0.000012
C	0.452747	−1.523416	0.000023
C	0.510902	−0.120912	0.000030
N	1.687738	−2.049244	0.000066
C	1.908254	0.236215	0.000013
C	2.617647	−1.008275	0.000058
C	2.653492	1.426479	−0.000024
C	4.011586	−1.088507	0.000076
C	4.040240	1.349662	−0.000007
C	4.712292	0.109799	0.000044
O	−3.911806	−1.623931	−0.000173
O	−1.428837	−3.118130	0.000019
H	−1.226129	2.042600	2.186151
H	−1.224809	3.557920	1.263531
H	0.229152	2.544859	1.306681
H	−1.225886	2.042671	−2.186072
H	0.229297	2.544902	−1.306421
H	−1.224671	3.557961	−1.263404
H	1.910314	−3.036959	0.000088
H	−4.767546	0.819254	−0.000166
H	−3.317961	2.810541	−0.000060
H	5.796759	0.092814	0.000056
H	2.165313	2.394550	−0.000062
H	4.623126	2.264592	−0.000031
H	4.524869	−2.044397	0.000110
H	−3.391463	−2.457081	−0.000186

Table S66. Energies for *m*/*z* 264a.

Zero-point correction	0.271742 (Hartree/Particle)
Thermal correction to Energy	0.287716
Thermal correction to Enthalpy	0.288660
Thermal correction to Gibbs Free Energy	0.229445
Sum of electronic and zero-point Energies	−860.955449
Sum of electronic and thermal Energies	−860.939475
Sum of electronic and thermal Enthalpies	−860.938531
Sum of electronic and thermal Free Energies	−860.997747

Table S67. Cartesian coordinates for *m*/*z* 264b.

C	3.586233	0.247417	−0.189003
C	2.894258	1.552296	−0.239336
C	2.902267	−0.950903	−0.102008
C	1.497264	−0.855741	−0.069417
C	0.742814	0.349766	−0.133347
C	1.395156	1.554108	−0.261808
C	0.690155	2.865856	−0.429744
C	0.593099	−2.060985	0.014922
C	−0.723528	−1.476474	0.005493
C	−0.679300	−0.078085	−0.068545
N	−2.013994	−1.910964	0.077813
C	−2.022525	0.383700	−0.027400
C	−2.837777	−0.804782	0.060039
C	−2.671391	1.642715	−0.036530
C	−4.234274	−0.752094	0.121427
C	−4.048350	1.686900	0.027663
C	−4.822981	0.501121	0.102404
O	3.545537	−2.119916	−0.047789
O	1.005232	−3.219243	0.073141
H	−0.163179	2.761470	−1.104155
H	1.350228	3.633308	−0.839108
H	0.309914	3.235068	0.531871
H	−2.312204	−2.873654	0.143205
H	4.672885	0.239741	−0.203003
H	−5.904117	0.580208	0.148847
H	−2.100939	2.561777	−0.085406
H	−4.555363	2.645719	0.022418
H	−4.830597	−1.656140	0.184212
H	2.886146	−2.846662	0.009536
C	3.459339	2.475667	0.907002
H	3.197590	2.064486	1.883606
H	4.545744	2.543320	0.827671
H	3.042156	3.479248	0.817180
H	3.230787	2.039225	−1.175254

Table S68. Energies for *m*/*z* 264b.

Zero-point correction	0.270290 (Hartree/Particle)
Thermal correction to Energy	0.286517
Thermal correction to Enthalpy	0.287461
Thermal correction to Gibbs Free Energy	0.227236
Sum of electronic and zero-point Energies	−860.932227
Sum of electronic and thermal Energies	−860.916001
Sum of electronic and thermal Enthalpies	−860.915057
Sum of electronic and thermal Free Energies	−860.975281

Table S69. Cartesian coordinates for *m*/*z* 246.

C	2.855356	1.379157	−0.050717
C	1.573494	1.449210	0.186256
C	1.038985	0.071743	0.265821
C	1.344022	−2.437029	0.354945
C	0.424850	2.446246	0.040747
C	−0.742501	1.571407	0.129016
C	−0.410768	0.214533	0.190519
N	−2.071440	1.745310	0.017722
C	−1.650463	−0.509573	0.044656
C	−2.672867	0.492845	−0.022624
C	−2.049104	−1.857814	−0.008240
C	−4.029336	0.189386	−0.119889
C	−3.396695	−2.159895	−0.115175
C	−4.377462	−1.151392	−0.166824
O	0.510579	3.631633	−0.125378
H	2.099673	−3.091826	0.787732
H	1.021336	−2.888919	−0.588948
H	0.498506	−2.419926	1.039854
H	−2.540158	2.633210	−0.105183
H	−5.421626	−1.428302	−0.244910
H	−1.326607	−2.659228	0.019709
H	−3.704974	−3.197069	−0.163350
H	−4.780756	0.969008	−0.154696
C	4.202032	−2.031264	−0.308466
H	3.826903	−2.740355	−1.052423
H	5.190704	−1.702798	−0.625486
H	4.305516	−2.575088	0.635662
C	1.885914	−1.046192	0.136652
C	3.811262	0.452795	−0.159698
H	4.870734	0.608810	0.005308
C	3.255979	−0.870455	−0.176029

Table S70. Energies for *m*/*z* 246.

Zero-point correction	0.240256 (Hartree/Particle)
Thermal correction to Energy	0.255552
Thermal correction to Enthalpy	0.256496
Thermal correction to Gibbs Free Energy	0.198310
Sum of electronic and zero-point Energies	−784.447408
Sum of electronic and thermal Energies	−784.432112
Sum of electronic and thermal Enthalpies	−784.431168
Sum of electronic and thermal Free Energies	−784.489354

1.7. Fragments for CQ3

m/*z* 364 corresponds to **CQ3_O1**.

Table S71. Cartesian coordinates for *m*/*z* 346.

C	−4.529844	−0.714620	−0.000001
C	−3.229427	−0.916455	0.000151
C	−2.691511	0.441933	0.000152
C	−2.908363	2.989951	−0.000032
C	−2.439780	−2.217951	0.000283
C	−1.139599	0.509688	0.000370
C	−1.020098	−1.992269	0.000192
C	−0.407963	−0.738626	0.000216
N	−0.066861	−2.966721	0.000079
C	1.006668	−0.964440	0.000061
C	1.182519	−2.382300	−0.000010
C	2.131619	−0.122248	−0.000005
C	2.451153	−2.977682	−0.000149
C	3.387900	−0.712933	−0.000144
C	3.539147	−2.126017	−0.000216
O	−3.009883	−3.293354	0.000159
O	−0.604432	1.604644	0.000031
H	−2.270935	3.142912	0.873363
H	−3.685435	3.750783	−0.000224
H	−2.270641	3.142776	−0.873237
H	−0.270076	−3.957731	0.000058
H	4.549905	−2.518485	−0.000323
H	2.022246	0.954355	0.000054
H	2.575143	−4.055223	−0.000202
C	−5.871004	2.652539	−0.000369
H	−5.713061	3.276487	−0.885738
H	−6.910489	2.322831	−0.000458
H	−5.713251	3.276607	0.884950
C	−3.501885	1.599896	−0.000022
C	−5.517402	0.177822	−0.000167
H	−6.584770	−0.002728	−0.000273
C	−4.924533	1.475810	−0.000187
C	4.649162	0.098752	−0.000221
O	5.756963	−0.398052	−0.000384
O	4.406781	1.418701	−0.000069
C	5.580501	2.279943	−0.000058
H	6.180372	2.042819	0.883019

H	6.179948	2.043393	−0.883579
C	5.093837	3.714206	0.000514
H	4.492285	3.925455	0.889011
H	5.952945	4.391275	0.000509
H	4.491833	3.926016	−0.887543

Table S72. Energies for *m*/*z* 346.

Zero-point correction	0.321124 (Hartree/Particle)
Thermal correction to Energy	0.344111
Thermal correction to Enthalpy	0.345055
Thermal correction to Gibbs Free Energy	0.267718
Sum of electronic and zero-point Energies	−1164.912696
Sum of electronic and thermal Energies	−1164.889709
Sum of electronic and thermal Enthalpies	−1164.888765
Sum of electronic and thermal Free Energies	−1164.966102

Table S73. Cartesian coordinates for *m*/*z* 336.

C	4.192865	−2.254885	0.000053
C	2.947988	−2.774889	0.000111
C	4.360606	−0.820317	0.000060
C	3.235211	−0.005280	0.000126
C	1.922396	−0.502141	0.000193
C	1.662218	−1.978263	0.000191
C	0.851129	−2.363910	1.277237
C	0.850987	−2.363875	−1.276776
C	3.225810	1.482441	0.000151
C	1.773657	1.793288	0.000186
C	1.011761	0.614342	0.000193
N	0.976398	2.873137	0.000229
C	−0.373506	1.015798	0.000176
C	−0.353158	2.448131	0.000221
C	−1.619710	0.368430	0.000139
C	−1.513519	3.224703	0.000239
C	−2.775608	1.138410	0.000156
C	−2.725433	2.547806	0.000207
O	5.585364	−0.335166	−0.000010
O	4.204251	2.212439	0.000182
H	1.410457	−2.131401	2.186314
H	0.640277	−3.436399	1.263694
H	−0.098381	−1.825597	1.306844
H	1.410212	−2.131339	−2.185909
H	−0.098527	−1.825561	−1.306258
H	0.640137	−3.436364	−1.263241
H	1.285893	3.837157	0.000251
H	5.082763	−2.874622	−0.000003
H	2.823134	−3.854723	0.000103
H	−3.651239	3.112820	0.000219
H	−1.690380	−0.713460	0.000101
H	−1.470662	4.308838	0.000273
H	5.559847	0.646794	−0.000023
C	−4.147743	0.439234	0.000122
O	−5.205847	1.120399	0.000137

O	−4.219723	−0.988953	0.000073
C	−5.590020	−1.397840	0.000042
H	−6.076491	−1.014841	−0.872630
H	−6.075552	−1.018121	0.874669
C	−5.666765	−2.935924	−0.002803
H	−5.180380	−3.318918	0.869920
H	−6.692091	−3.241874	−0.002926
H	−5.181146	−3.315648	−0.877379

Table S74. Energies for *m*/*z* 336.

Zero-point correction	0.342709 (Hartree/Particle)
Thermal correction to Energy	0.364716
Thermal correction to Enthalpy	0.365660
Thermal correction to Gibbs Free Energy	0.291322
Sum of electronic and zero-point Energies	−1128.085892
Sum of electronic and thermal Energies	−1128.063886
Sum of electronic and thermal Enthalpies	−1128.062941
Sum of electronic and thermal Free Energies	−1128.137280

Table S75. Cartesian coordinates for *m*/*z* 292.

C	4.003336	−1.347698	−0.000107
C	2.988301	−2.236420	−0.000049
C	3.706399	0.065792	−0.000100
C	2.380305	0.480853	−0.000034
C	1.293500	−0.407521	0.000033
C	1.516002	−1.889790	0.000031
C	0.869554	−2.513243	1.277077
C	0.869409	−2.513256	−1.276936
C	1.898518	1.888433	−0.000009
C	0.422870	1.721591	0.000026
C	0.075215	0.361615	0.000033
N	−0.676275	2.492030	0.000069
C	−1.365816	0.301943	0.000016
C	−1.801792	1.666464	0.000061
C	−2.341626	−0.707960	−0.000021
C	−3.148813	2.033941	0.000079
C	−3.682319	−0.345315	−0.000004
C	−4.082725	1.006939	0.000047
O	4.713436	0.915074	−0.000170
O	2.594187	2.891573	0.000022
H	1.325973	−2.115009	2.186154
H	1.010529	−3.597133	1.263534
H	−0.201818	−2.304650	1.306684
H	1.325720	−2.115028	−2.186069
H	−0.201968	−2.304662	−1.306418
H	1.010385	−3.597144	−1.263401
H	−0.689245	3.504430	0.000091
H	5.044069	−1.652440	−0.000163
H	3.213149	−3.299939	−0.000057
H	−5.140109	1.248384	0.000059
H	−2.064751	−1.756207	−0.000059
H	−3.452771	3.075476	0.000113

H	4.377125	1.837999	−0.000183
C	−4.761062	−1.444366	−0.000038
O	−5.980803	−1.134846	−0.000023
H	−4.472459	−2.474710	−0.000074

Table S76. Energies for *m*/*z* 292.

Zero-point correction	0.280769 (Hartree/Particle)
Thermal correction to Energy	0.298820
Thermal correction to Enthalpy	0.299764
Thermal correction to Gibbs Free Energy	0.235606
Sum of electronic and zero-point Energies	−974.263182
Sum of electronic and thermal Energies	−974.245131
Sum of electronic and thermal Enthalpies	−974.244187
Sum of electronic and thermal Free Energies	−974.308345

1.8. Fragments for CQ4

Table S77. Cartesian coordinates for *m*/*z* 342.

C	−4.360974	−1.706516	0.000074
C	−3.238051	−2.454741	0.000094
C	−4.251554	−0.266771	0.000117
C	−2.990313	0.318940	0.000178
C	−1.797810	−0.420332	0.000202
C	−1.823917	−1.918582	0.000165
C	−1.101433	−2.452709	−1.276902
C	−1.101541	−2.452778	1.277263
C	−2.698236	1.777585	0.000271
C	−1.213381	1.805913	0.000179
C	−0.691415	0.502817	0.000133
N	−0.223496	2.713629	0.000159
C	0.744722	0.633438	0.000005
C	0.997952	2.043041	0.000053
C	1.837264	−0.245758	−0.000137
C	2.288734	2.577443	−0.000013
C	3.349961	1.685896	−0.000142
O	−5.360011	0.443686	0.000076
O	−3.519805	2.680687	0.000344
H	−1.604930	−2.116737	−2.186239
H	−1.100175	−3.545638	−1.263788
H	−0.065965	−2.107470	−1.305711
H	−1.605116	−2.116857	2.186576
H	−0.066077	−2.107545	1.306183
H	−1.100283	−3.545707	1.264090
H	−0.342814	3.719151	0.000203
H	−5.352873	−2.144834	0.000020
H	−3.322212	−3.538499	0.000055
H	4.369334	2.052892	−0.000200
H	1.708342	−1.320817	−0.000191
H	2.461934	3.648429	0.000026
H	−5.147635	1.402917	0.000063
C	3.117482	0.293823	−0.000205
Br	4.610472	−0.874112	−0.000388

Table S78. Energies for *m*/*z* 342.

Zero-point correction	0.261413 (Hartree/Particle)
Thermal correction to Energy	0.278938
Thermal correction to Enthalpy	0.279882
Thermal correction to Gibbs Free Energy	0.215928
Sum of electronic and zero-point Energies	−3432.064275
Sum of electronic and thermal Energies	−3432.046750
Sum of electronic and thermal Enthalpies	−3432.045806
Sum of electronic and thermal Free Energies	−3432.109760

Table S79. Cartesian coordinates for *m*/*z* 291.

C	4.009899	0.730976	−0.000233
C	3.909560	−0.617993	−0.000012
C	2.815050	1.508065	−0.000325
C	1.508412	0.867862	−0.000162
C	1.409530	−0.502282	0.000041
C	2.631012	−1.384848	0.000135
C	2.646871	−2.298859	1.280106
C	2.646801	−2.299249	−1.279563
C	0.312152	1.773983	−0.000259
C	0.023431	−1.161725	0.000408
C	−0.959194	1.121115	−0.000060
C	−1.115371	−0.264285	0.000182
N	−2.186195	1.721217	0.000017
C	−2.518731	−0.528965	0.000092
C	−3.165677	0.751595	0.000041
C	−3.300890	−1.708495	0.000129
C	−4.562581	0.899630	0.000014
C	−4.649142	−1.494322	0.000105
C	−5.318874	−0.266832	0.000043
O	2.917885	2.794830	−0.000603
O	0.451796	3.014474	−0.000051
O	−0.084020	−2.383486	0.000004
H	2.643051	−1.698224	2.192965
H	3.554134	−2.907870	1.269023
H	1.777602	−2.954113	1.268781
H	2.642888	−1.698890	−2.192604
H	1.777561	−2.954536	−1.267972
H	3.554087	−2.908224	−1.268368
H	−2.330857	2.722258	−0.000040
H	4.959039	1.255268	−0.000359
H	4.815809	−1.218873	0.000065
H	−5.031809	1.878186	−0.000025
H	−6.403214	−0.218379	0.000034
H	−2.840975	−2.690148	0.000208
H	1.967222	3.186252	−0.000717

Table S80. Energies for *m*/*z* 291.

Zero-point correction	0.268206 (Hartree/Particle)
Thermal correction to Energy	0.285494
Thermal correction to Enthalpy	0.286439
Thermal correction to Gibbs Free Energy	0.223525
Sum of electronic and zero-point Energies	−973.611699
Sum of electronic and thermal Energies	−973.594411
Sum of electronic and thermal Enthalpies	−973.593467
Sum of electronic and thermal Free Energies	−973.656381

Table S81. Cartesian coordinates for *m*/*z* 263.

C	−3.666835	0.681423	−0.000110
C	−2.879464	1.776946	−0.000052
C	−3.057473	−0.628081	−0.000103
C	−1.671832	−0.732009	−0.000037
C	−0.814478	0.379430	0.000030
C	−1.366917	1.772785	0.000028
C	−0.878474	2.526451	1.277074
C	−0.878334	2.526496	−1.276939
C	−0.883762	−1.993892	−0.000012
C	0.515750	−1.497166	0.000023
C	0.546350	−0.093790	0.000030
N	1.760830	−1.998638	0.000066
C	1.936419	0.290711	0.000013
C	2.670116	−0.939607	0.000058
C	2.658138	1.495381	−0.000024
C	4.065362	−0.992448	0.000076
C	4.046127	1.445813	−0.000007
C	4.742399	0.219388	0.000044
N	−3.845987	−1.683377	−0.000173
N	−1.334152	−3.128525	0.000019
H	−1.232835	2.035191	2.186151
H	−1.261275	3.550245	1.263531
H	0.212301	2.565933	1.306681
H	−1.232594	2.035267	−2.186072
H	0.212445	2.565979	−1.306421
H	−1.261138	3.550288	−1.263404
H	2.002761	−2.981792	0.000088
H	−4.749544	0.742531	−0.000166
H	−3.339345	2.761902	−0.000060
H	5.826990	0.223704	0.000056
H	2.151041	2.453678	−0.000062
H	4.597319	−1.938074	0.000110
H	−3.309382	−2.506147	−0.000186

Table S82. Energies for *m*/*z* 263.

Zero-point correction	0.258601 (Hartree/Particle)
Thermal correction to Energy	0.274543
Thermal correction to Enthalpy	0.275487
Thermal correction to Gibbs Free Energy	0.215710
Sum of electronic and zero-point Energies	−860.276607
Sum of electronic and thermal Energies	−860.260665
Sum of electronic and thermal Enthalpies	−860.259720
Sum of electronic and thermal Free Energies	−860.319498

2. Mass Spectra

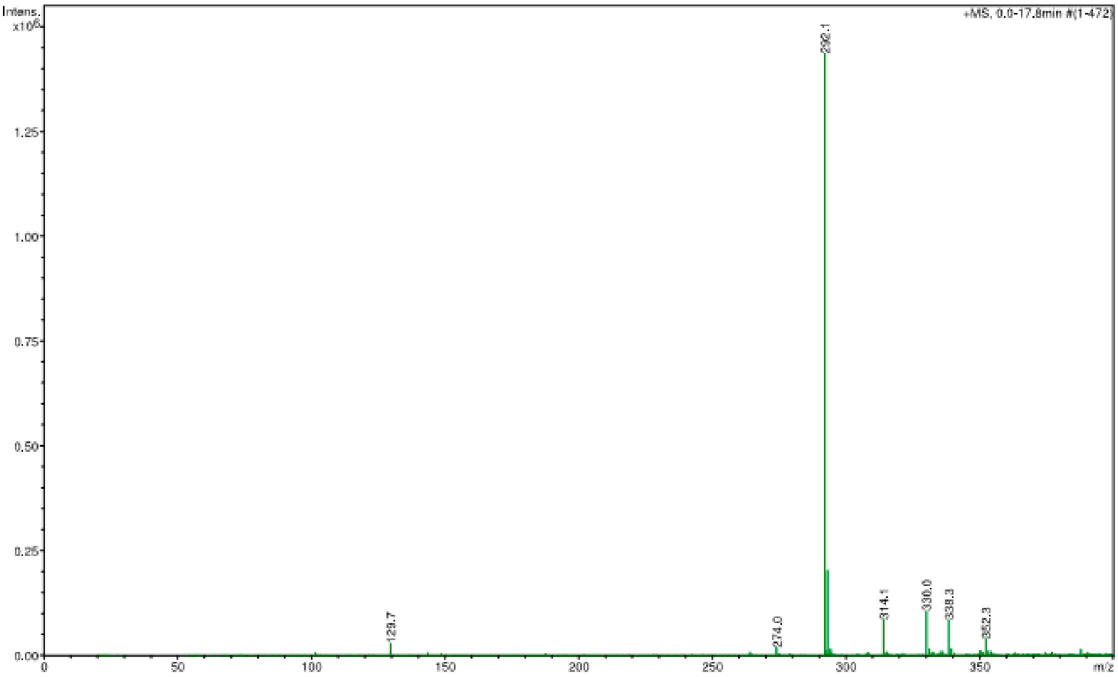

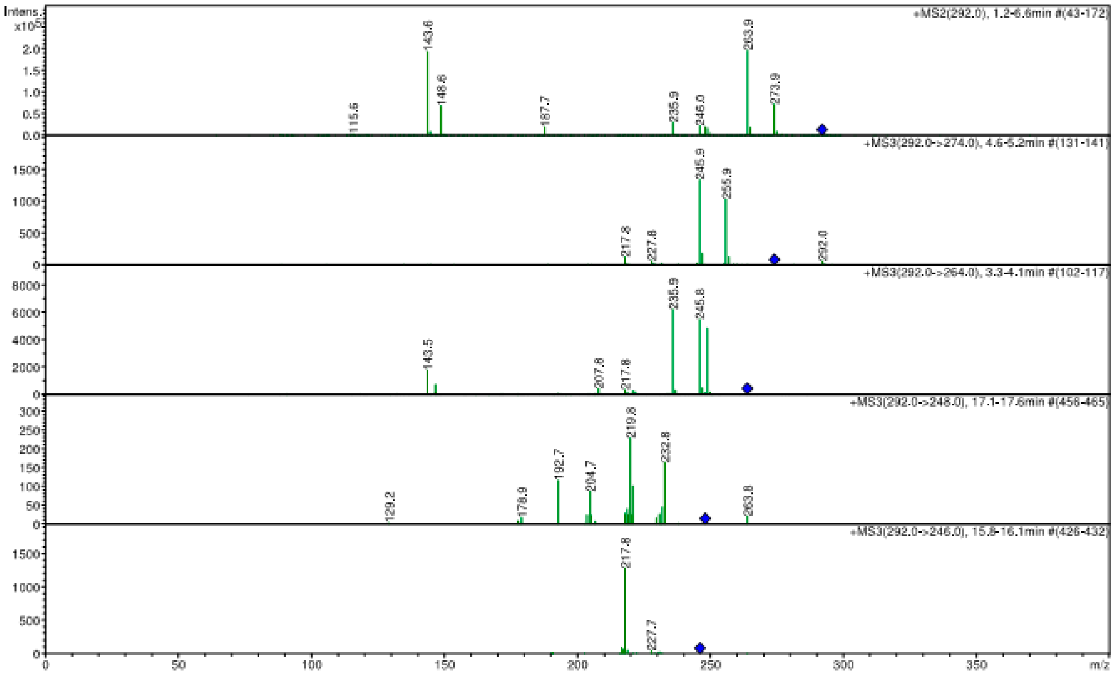

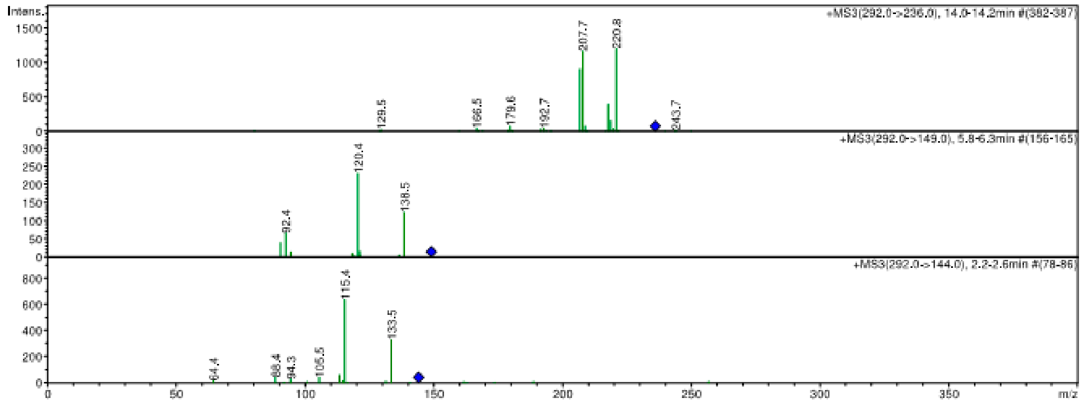

Figure S1. Mass spectrum for **CQ1**.

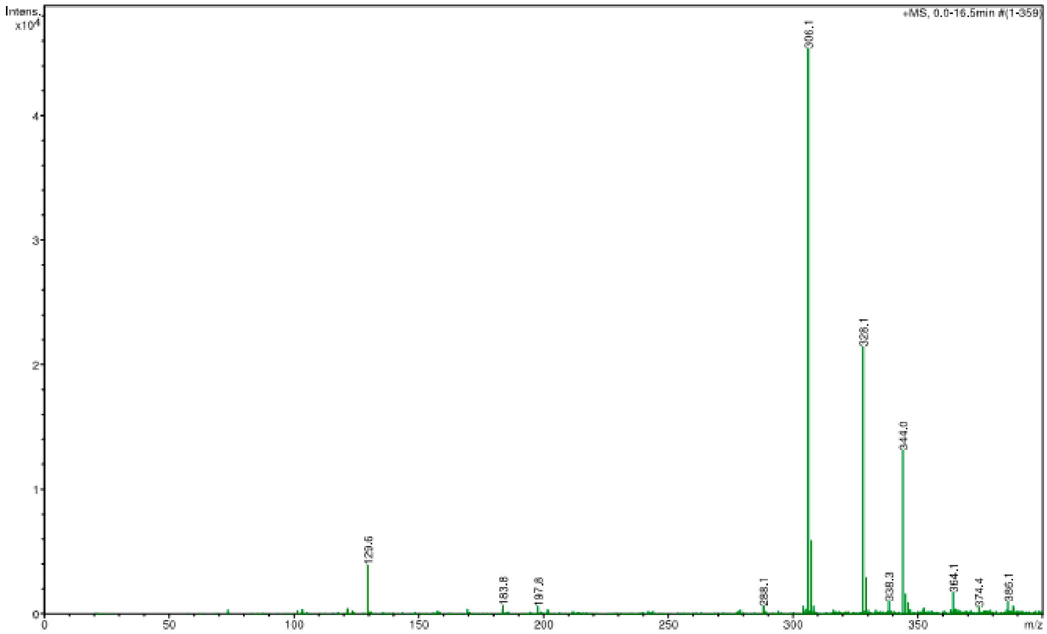

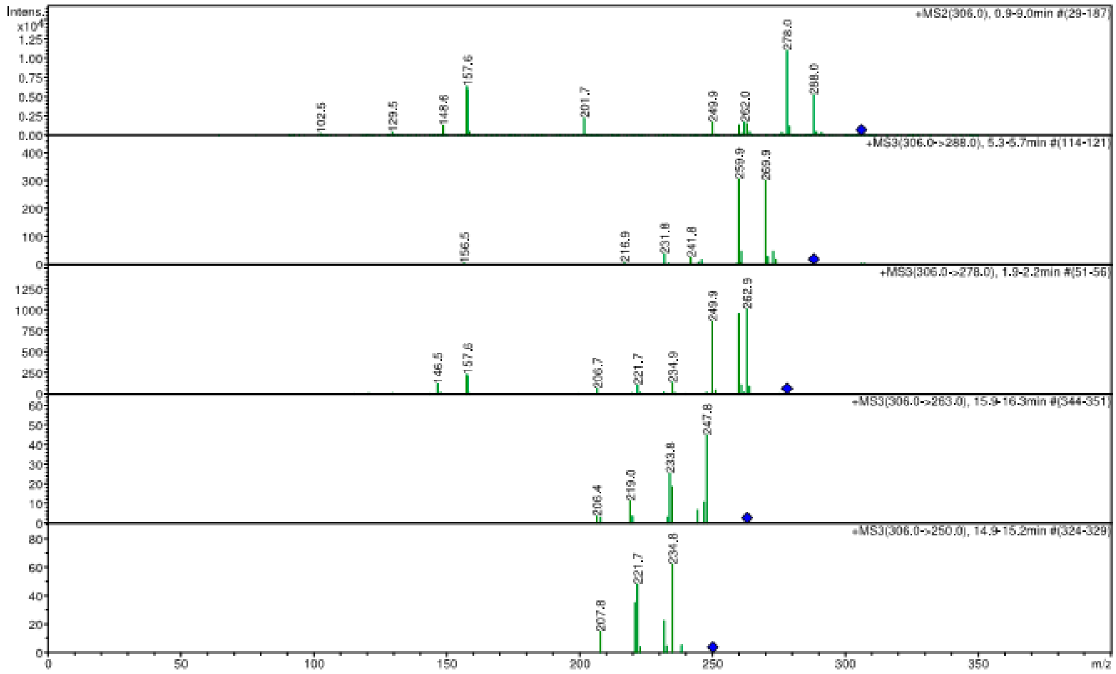

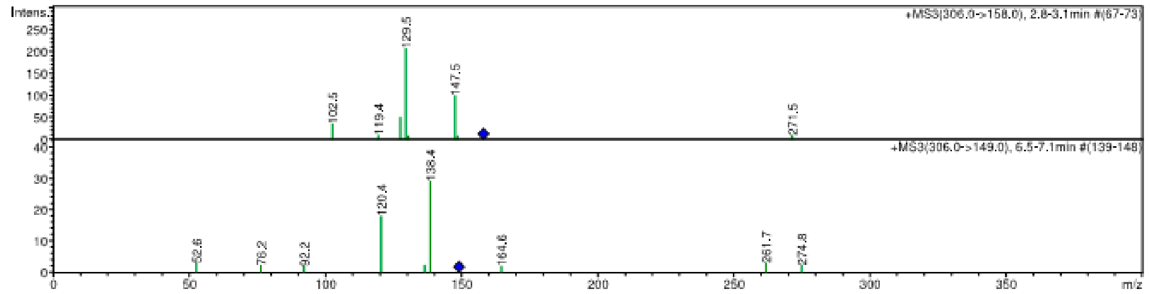

Figure S2. Mass spectrum for **CQ2**.

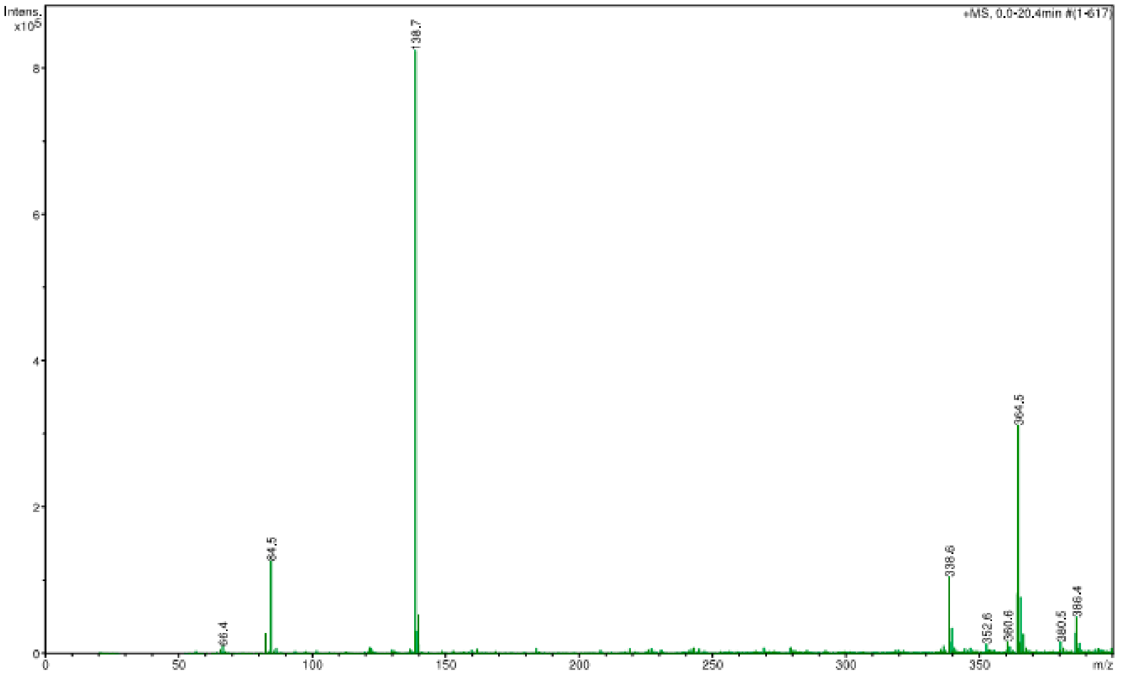

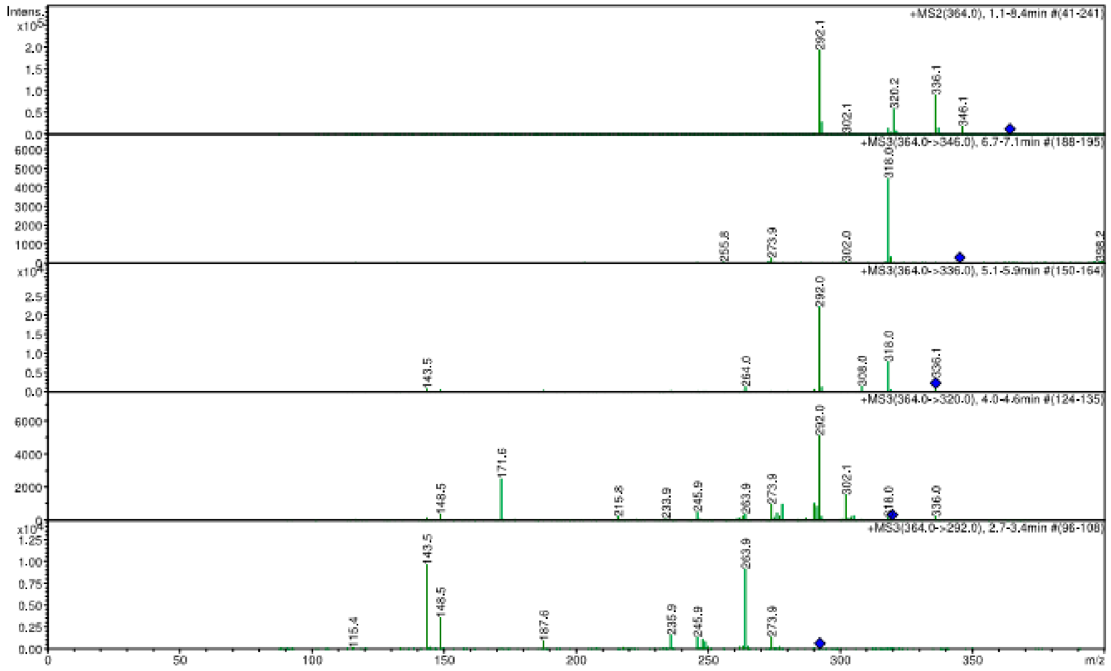

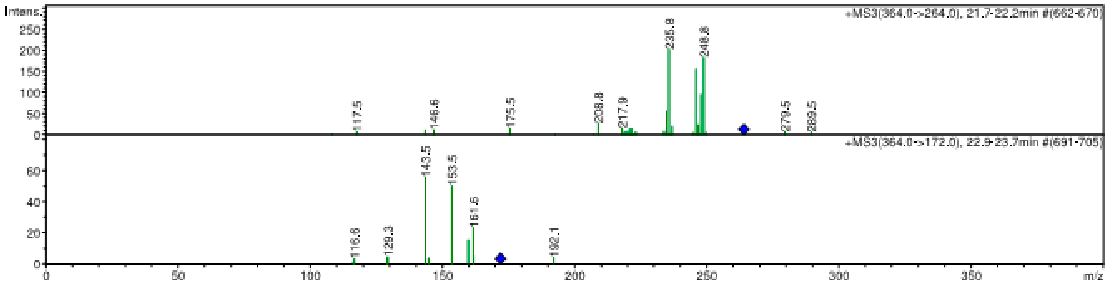

Figure S3. Mass spectrum for **CQ3**.

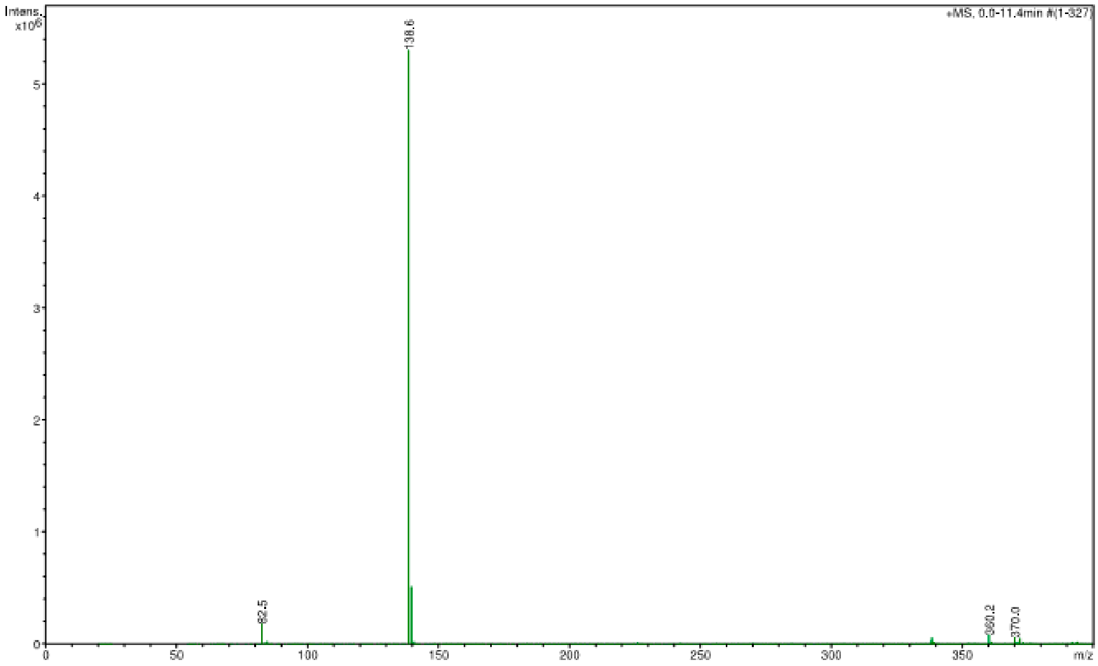

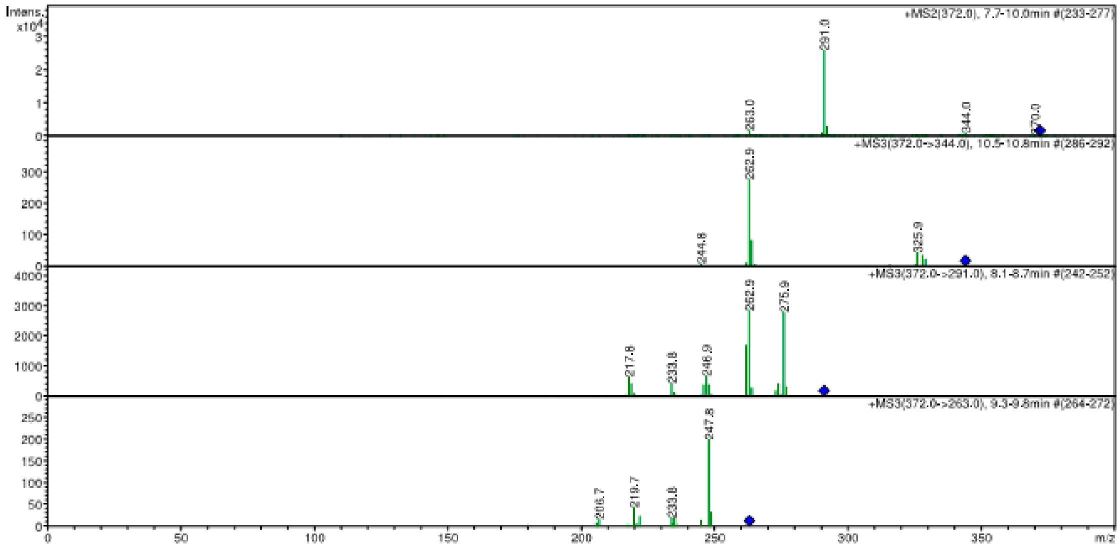

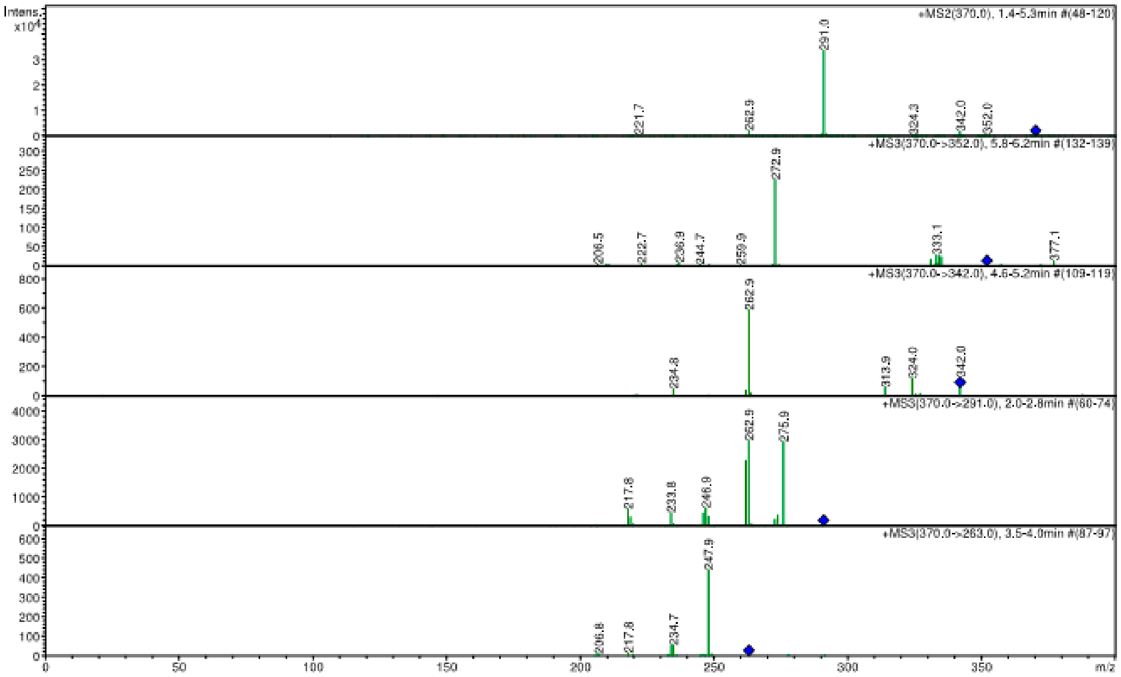

Figure S4. Mass spectra for **CQ4**.

3. Proton Affinity and Gas Phase Basicity of CQ1–CQ4 for all protonation sites studied.

Table S83. Proton Affinity (PA) and Gas Phase Basicity (GB) for O1.

Compound	PA	GB
**CQ1**	240.0	231.3
**CQ2**	241.0	232.4
**CQ3**	238.4	229.9
**CQ4**	238.1	229.5

Table S84. Proton Affinity (PA) and Gas Phase Basicity (GB) for O2.

Compound	PA	GB
**CQ1**	240.0	231.2
**CQ2**	241.0	232.4
**CQ3**	238.4	229.9
**CQ4**	238.1	229.5

Table S85. Proton Affinity (PA) and Gas Phase Basicity (GB) for O3.

Compound	PA	GB
**CQ1**	214.3	207.1
**CQ2**	215.9	209.0
**CQ3**	212.8	205.5
**CQ4**	211.8	204.5

Table S86. Proton Affinity (PA) and Gas Phase Basicity (GB) for N4.

Compound	PA	GB
**CQ1**	193.7	186.5
**CQ2**	195.5	188.6
**CQ3**	191.5	184.4
**CQ4**	190.3	183.0

Table S87. Proton Affinity (PA) and Gas Phase Basicity (GB) for O5.

Compound	PA	GB
**CQ1**	/	/
**CQ2**	/	/
**CQ3**	212.8	205.2
**CQ4**	/	/

Table S88. Proton Affinity (PA) and Gas Phase Basicity (GB) for O6.

Compound	PA	GB
**CQ1**	/	/
**CQ2**	/	/
**CQ3**	194.7	188.7
**CQ4**	/	/
